# Training Emotion Recognition Accuracy: Results for Multimodal Expressions and Facial Micro Expressions

**DOI:** 10.3389/fpsyg.2021.708867

**Published:** 2021-08-12

**Authors:** Lillian Döllinger, Petri Laukka, Lennart Björn Högman, Tanja Bänziger, Irena Makower, Håkan Fischer, Stephan Hau

**Affiliations:** ^1^Department of Psychology, Faculty of Social Sciences, Stockholm University, Stockholm, Sweden; ^2^Department of Psychology and Social Work, Mid Sweden University, Sundsvall, Sweden; ^3^Evidens University College, Göteborg, Sweden

**Keywords:** emotion recognition, emotion recognition training, multimodal emotion recognition, micro expression recognition, nonverbal communication

## Abstract

Nonverbal emotion recognition accuracy (ERA) is a central feature of successful communication and interaction, and is of importance for many professions. We developed and evaluated two ERA training programs—one focusing on dynamic multimodal expressions (audio, video, audio-video) and one focusing on facial micro expressions. Sixty-seven subjects were randomized to one of two experimental groups (multimodal, micro expression) or an active control group (emotional working memory task). Participants trained once weekly with a brief computerized training program for three consecutive weeks. Pre-post outcome measures consisted of a multimodal ERA task, a micro expression recognition task, and a task about patients' emotional cues. Post measurement took place approximately a week after the last training session. Non-parametric mixed analyses of variance using the Aligned Rank Transform were used to evaluate the effectiveness of the training programs. Results showed that multimodal training was significantly more effective in improving multimodal ERA compared to micro expression training or the control training; and the micro expression training was significantly more effective in improving micro expression ERA compared to the other two training conditions. Both pre-post effects can be interpreted as large. No group differences were found for the outcome measure about recognizing patients' emotion cues. There were no transfer effects of the training programs, meaning that participants only improved significantly for the specific facet of ERA that they had trained on. Further, low baseline ERA was associated with larger ERA improvements. Results are discussed with regard to methodological and conceptual aspects, and practical implications and future directions are explored.

## Introduction

Nonverbal emotion recognition accuracy (ERA)—the ability to correctly infer another person's emotional state from their nonverbal behavior, including facial expressions, bodily postures and voice—is a crucial part of social interactions. Expressing emotions nonverbally is a fast way of explicitly and implicitly communicating inner states, and the ability to read and understand another person's inner states provides an advantage in everyday relationships as well as in professional settings. Even though healthy adults are relatively accurate at recognizing others people's emotions, there are important individual differences in ERA, and low ERA is associated with personal and interpersonal drawbacks (see e.g., Hall et al., 2009; Schlegel et al., 2017). Emotion recognition training has been shown to improve ERA (for reviews, see e.g., Blanch-Hartigan and Ruben, 2013; Rebeschini et al., 2019), and first evidence even suggests positive real-life outcomes of ERA training (Schlegel, 2021). However, existing ERA trainings often suffer from limited ecological validity due to the predominant use of static facial items and other methodological limitations. Two computerized self-administered ERA training programs for healthy adult populations are evaluated in the present study. One focuses on dynamic facial, vocal, and multimodal expressions, and the other on facial micro expressions. In the following, previous studies on ERA training are reviewed and the contribution of the present study toward bridging scientific and methodological gaps will be outlined.

### Emotion Recognition Accuracy

Research on ERA reports individual differences in the ability to recognize nonverbal expressions of emotion in others. ERA is generally associated with psychological health and well-being (Bänziger, 2016). Meta-analyses report, for example, negative associations between ERA and depression (Dalili et al., 2015) and antisocial traits (Marsh and Blair, 2008). Hall et al. (2009) conducted a meta-analysis about psychosocial correlates of interpersonal sensitivity (also called *interpersonal accuracy;* Hall et al., 2016b)—an umbrella term for judging other people's emotions, personality traits, thoughts and feelings. They found positive correlations with traits such as empathy, extraversion, conscientiousness, and openness, as well as better social competencies and positive adjustment; and negative associations with shyness, neuroticism, depression, and other negative personality traits. Wang et al. (2019) found that ERA is associated with peer status and friendship quality in school children. The literature about ERA and work-related outcomes draws a diverse picture. The review by Bechtoldt et al. (2019) concluded that the evidence about ERA and work outcomes is inconclusive, citing studies that find positive, non-existent or negative associations. Based on a meta-analysis, Joseph and Newman (2010) report that the relationship between ability-based emotional intelligence (e.g., emotion recognition) and job performance is inconsistent and likely depending on service sector.

However, ERA also varies greatly depending on methodological choices, such as test item preselection, channel of expression (face, voice, body), or type of emotion (Hall et al., 2009; Bänziger, 2016). Generally, still pictures of stereotypical emotions have higher ERA rates than dynamic stimuli (Khosdelazad et al., 2020), auditory ERA is more difficult than visual or audio-visual ERA (Cortes et al., 2021) and there seems to be a ceiling effect for the detection of happiness, which is likely due to the fact that happiness often has been the only available positive emotion in ERA tests (see e.g., Kessels et al., 2014).

### Training Emotion Recognition Accuracy

The research on explicit training of ERA is largely targeting individuals with psychopathologies that are associated with socio-emotional perception deficits, with the aim of helping them understand social communication more efficiently, and thereby increasing societal participation and quality of life. There are various ERA training programs for individuals with schizophrenia or autism spectrum disorder that led to increased ERA and related socio-emotional abilities (for reviews see e.g., Bordon et al., 2017; Berggren et al., 2018). Further, training could improve ERA for example in patients with chronic post-traumatic brain injury (Neumann et al., 2015), young offenders (Hubble et al., 2015), women with Anorexia Nervosa (Preis et al., 2020), adults with intellectual disabilities (Rydin-Orwin et al., 1999), and children with developmental delays (Downs and Strand, 2008). Some of the studies also found outcomes such as reduction of subsequent crimes in young offenders (Hubble et al., 2015), or reduction of Anorexia Nervosa symptoms (Preis et al., 2020). There are also large-scale school or preschool interventions to strengthen children's socio-emotional competencies (e.g., Brackett et al., 2012).

Non-clinical adult populations could also profit from ERA training, such as individuals working in professions that rely on socio-emotional perception and communication. For example, health care providers are confronted with patients' strong emotions on a daily basis and need to draw inferences from patients' verbal and nonverbal emotional expressions to be able to provide good care. Psychotherapists could profit from ERA training to get a better understanding of their clients' inner worlds, to facilitate a good therapeutic relationship and to choose appropriate interventions (for a discussion of potential benefits see e.g., Hutchison and Gerstein, 2012). Service provider jobs in which customer satisfaction and customer loyalty are essential, such as salespeople or waiters, have to be able to read customers' emotional expressions and to respond to them in satisfactory ways (Bechtoldt et al., 2019). Law enforcement and security personnel need to detect and react to potential threats by other individuals fast and accurately, oftentimes based on limited social information, to protect themselves and others from harm (Hurley, 2012).

Apart from professional benefits, increased ERA has been shown to be related to positive personality traits that are beneficial for social interaction and interpersonal relationships (Hall et al., 2009). The ability to accurately perceive how another person feels is fundamental for many other inner and interpersonal socio-emotional processes (see e.g., Joseph and Newman, 2010), such as emotion regulation and empathy, and could thus be considered relevant for all social encounters. Surprisingly, the literature about ERA training for healthy adults is sparse, especially for randomized controlled studies. In the following, articles and review articles about ERA training for healthy adult populations are presented. Training studies for general ERA and micro expression ERA are reported separately.

### Review of Emotion Recognition Trainings for Healthy Adults

As one of the first, Elfenbein (2006) investigated ERA training in a cross-cultural context using still pictures of faces and providing feedback about the intended emotion and difficulty level. She found that the ERA improvement for faces belonging to another cultural group than one's own was greatest, hypothesizing that this training provided more novel information. However, the study did not include comparison to a control group. A review by Blanch-Hartigan and Ruben (2013) included five experimental studies about ERA trainings tailored to health care professionals (published until 2012). Blanch-Hartigan (2012) investigated different ERA training conditions (raising consciousness, instruction, practice alone, practice with feedback, and a comprehensive condition including all components) for training clinicians to recognize patients' verbal and nonverbal cues and found that practice alone and practice with feedback, as well as the comprehensive training successfully increased ERA in comparison to a control group (in a sample of undergraduate students). Computerized practice with feedback was found to be the most effective component. It needs to be noted, though, that the training and test did not exclude verbal content, thus, the study does not provide evidence for nonverbal ERA *per se*. Riess et al. (2012) reported that a group of medical residents showed a significant improvement in ERA after receiving a three session (á 60 min) empathy and relational skills group training including instruction on facial expressions of emotion, compared to those that only received standard residency or fellowship training. They also reported an improvement in patient-rated empathy for the participants in the training group. Two studies found no significant increase in ERA of medical residents after empathy and relational skills training (3 sessions á 90 min) using videos of difficult patient-physician interactions (Riess et al., 2011), or an interpersonal process recall training using videotapes of themselves in interaction with clients (Robbins et al., 1979), respectively. The fifth study concerned a micro expression recognition training for medical students and is discussed below (Endres and Laidlaw, 2009).

In a systematic review about facial expression recognition training for adults, Rebeschini et al. (2019) reviewed studies from the years 2013–2018 in which they found 26 articles about training programs for adults with psychopathologies, and nine articles about training for healthy adults (presented in the following). Huelle et al. (2014) demonstrated that female participants significantly increased their ERA by watching and categorizing dynamic facial emotional displays and found consolidation of ERA in a second training session 2 days to several weeks after the first training. However, there was no comparison to a control group and no active training component including feedback, only prolonged exposure to items. Pollux et al. (2014) investigated gaze behavior changes due to facial expression training in a small sample of adults and children. The training consisted of four sessions on 4 consecutive days with a self-paced facial expression categorization task including feedback for fearful, happy and sad faces with varying intensity. Results showed that the adults' ERA for happy and sad faces improved for items within a certain intensity range and that improvements in ERA for sadness coincided with a bias toward gazing at the sender's eyes. In another small sample study, Pollux (2016) found large improvements in the ability to recognize subtle facial expressions due to three training sessions with morphed faces showing happy, sad or surprised faces, as well as event-related potential (ERP) modulations in the brain due to training. In a group intervention, Ragsdale et al. (2016) presented health care providers with information about facial expression features (i.e., typical muscle contractions) and facilitated three interactive exercises about facial emotional expressions in general and in the medical context. They found a substantial improvement from pre to posttest. However, the outcome measure was no standardized ERA test, but a quiz about facial expression features for specific emotions, and there was no control group, which limits the validity of the result.

Schlegel et al. (2017) evaluated a 35 min dynamic nonverbal audio-visual ERA training in four different samples using an audio-visual outcome measure. Training and test included (different) items from the Geneva Multimodal Emotion Portrayals Core Set (GEMEP; Bänziger et al., 2012). In contrast to most other studies which have focused on a small number of basic emotions, the authors investigated 14 emotions of which six were positive emotions and one neutral. They found that the audio-visual training significantly improved young to middle-aged participants' ERA compared to various control groups and that effect sizes were large. Further, they report small transfer effects to unimodal facial and vocal ERA tests using other stimulus material. Notably, the audio-visual training did not improve ERA in a sample of older adults. This study is, to our knowledge and until the present study, the only ERA training study to include nonverbal audio content. In a later study, Schlegel (2021) reported face-to-face interaction outcomes of the audio-visual training, demonstrating a causal link between ERA training and cooperativeness and other relational outcomes in a negotiation task.

In a study about emotion specificity in training ERA, Du et al. (2016) reported that 8 days (á 1h) of training happy or sad morphed faces with feedback led to improvements in recognition accuracy for the target emotion and that those effects lasted a month and transferred to other gender facial stimuli. In another study investigating specificity (Griffiths et al., 2015), participants trained the recognition of low intensity happy and fearful faces using morphed facial expressions and feedback. It was found that the increased accuracy for the target emotion disappeared when taking into account the increased false alarm rate for the target emotion. Even though methodologically different from other ERA studies, the results about specificity in ERA trainings provide evidence for possible bias in ERA training and the importance of taking into account false alarm rates in ERA tasks when establishing effectiveness of a training. Finally, two studies (Hurley et al., 2014; Yu et al., 2016) included in the review concerned micro expressions and are discussed below.

### Review of Micro Expression Trainings for Healthy Adults

Traditionally, a facial emotional expression is considered a micro expression when it is shorter than 200 ms, though more recent research proposes a more liberal cut-off of <500 ms (see Matsumoto and Hwang, 2018). Micro expression research is based on the idea that facial emotional expressions cannot fully be voluntarily controlled, and that even in cases where humans consciously or unconsciously try to squelch, neutralize, or mask their emotions (e.g., due to social desirability), very brief, involuntary facial expressions may leak through (Darwin, 1872). Research on micro expressions has consequently been strongly focused on *basic emotions* (usually anger, contempt, disgust, fear, sadness, happiness, and surprise; see e.g., Ekman and Cordaro, 2011). It is further argued that an untrained observer likely will not become consciously aware of micro expressions (see e.g., Ekman and Friesen, 1969; Porter and ten Brinke, 2008; Frank and Svetieva, 2015), which makes them an attractive candidate for ERA training. Ekman and colleagues offer a commercial micro expression ERA training, the *Micro Expression Training Tool* (Paul Ekman Group, 2020a), consisting of five parts (pretest, training with instruction about facial expressions for basic emotions, practice with feedback, review, posttest). Despite its broad commercial use, there are only a few peer-reviewed studies about Ekman et al.'s training or micro expression trainings in general.

Matsumoto and Hwang (2011) were the first to systematically research whether micro expressions could be trained. In two randomized controlled studies, they found significantly higher micro expression ERA immediately after Ekman et al.'s (Paul Ekman Group, 2020a) training as well as 2–3 weeks after training. Further, they reported better third-party ratings of social and communicative job skills 2 weeks after the training. Hurley (2012) compared three micro expression training conditions (using Ekman et al.'s training with varying degrees of instruction) to three control conditions in a student sample. Results showed that the training sessions were successful and that the instructor feedback plus descriptions training was the most effective. However, reinforcement at weeks 3 and 6 did not improve micro expression ERA. Investigating the same sample, Hurley et al. (2014) reported that young age and openness to experiences predicted micro expression ERA before training, however, after training, no individual difference variables predicted outcome. In a second study they administered Ekman et al.'s training to airport behavior detection officers (Hurley et al., 2014). Results showed that having no prior facial expression training, being younger, less confident in one's own micro expression ERA abilities, and being less conscientious predicted micro expression ERA improvement post training.

Yu et al. (2016) evaluated a group intervention about micro and subtle emotional expressions using Ekman et al.'s micro expression training and the *Subtle Expression Training Tool* (Paul Ekman Group, 2020b) in a randomized controlled trial using a sample of medical students. The intervention (1 h) is briefly described as containing exercises using pictures of facial affects, and written and video information (i.e., not using all training components). They reported that both trainings significantly improved ERA and state large effect sizes. Another study (Endres and Laidlaw, 2009) included medical students and investigated moderators for micro expression ERA trainability. They found that participants with high communicative skills became significantly more accurate in detecting facial micro expressions after undergoing the Ekman et al.'s (Paul Ekman Group, 2020a) micro expression training, in contrast to their colleagues with low communicative skills that did not profit from training.

Although not the focus of the current study, it should be noted that micro expression training has also been used as a tool for spotting nonverbal indicators for deception, but this purpose has been criticized due to the number of false positives and negatives, and the consequences thereof (Weinberger, 2010; see also Burgoon, 2018; Vrij et al., 2019). Porter and ten Brinke (2008) examined the relationship between micro expressions and deception, and their results suggested that micro expressions may not be as frequent as previously thought. Only about 22% of the participants displayed micro expressions and only in either the upper or the lower half of the face, and only 2% of all expressions analyzed were micro expressions. Importantly, micro expressions did not occur more frequently in deceptive scenarios, although the frequency of deception detection due to inconsistent expressions was significantly above chance. Zloteanu et al. (2021) found a generally high micro expression ERA among their participants, but this was not related to accuracy in judging true or false statements and there was no effect of micro expression training on deception detection. Jordan et al. (2019) likewise found no evidence that micro expression training improves detection of deception. In a comprehensive review of the field, Vrij et al. (2019) thus argue that many nonverbal indicators of deception lack scientific support and instead suggest a stronger focus on verbal indicators of deception. Further, ethical implications of micro expression detection procedures in law enforcement and security contexts cannot be disregarded (see e.g., Weinberger, 2010).

### Summary and Present Study

Most studies found positive effects for their respective ERA training, though publication bias could contribute to this finding. Rebeschini et al. (2019) report 91.4% positive results and effect sizes varying from small to very large. Still, the existing studies about ERA training for healthy adult populations vary greatly in type of intervention and methodology, such as length and quality of training, individual or group administration, computer or other administration, or ERA facets trained (e.g., static facial emotional expressions, facial micro expressions, dynamic emotional expressions). The vast majority of studies investigate basic emotions and facial emotional expressions; there was only one study incorporating nonverbal audio-visual ERA and more diverse emotions (Schlegel et al., 2017). Some training programs target specific populations such as health care professionals; most studies used student samples. Limitations include assessing outcome with the same instrument/items as used for the training, absence of a control group, absence of pretest ERA, conduction of training and posttest in immediate succession, or attempting to increase ERA by training related constructs such as empathy or interpersonal skills, all of which reduce the validity and generalizability of the findings. Several studies also did not report descriptive ERA data or effect sizes.

The present study seeks to overcome the identified limitations found in ERA training research, and aims to develop and evaluate new and more ecologically valid computerized ERA training programs that could be useful in different professional contexts, as well as for the general public. Following the literature, we decided to develop one multimodal ERA training and one micro expression ERA training, two related but not identical emotion recognition competencies relevant for many kinds of social encounters. First, we decided to use dynamic items to assess audio-visual ERA, which is arguably more ecologically valid than the use of stereotypical static faces (Schlegel et al., 2017). Additionally, we decided to also train the audio and video modalities separately (unimodally), to investigate these complementary nonverbal ERA modalities more systematically. To our knowledge, this is the first training study investigating nonverbal auditive ERA unimodally. Further, we extended the number of emotional expressions to 12 more diverse emotions, to capture more complex and interpersonal emotions (such as pleasure, irritation or relief), and to include multiple positive emotions to capture a more realistic array of positive emotions, as well as to prevent a ceiling effect for happiness. Finally, the multimodal training and outcome measure provide the opportunity to investigate the trainability of recognizing emotional expressions with varying valence and arousal levels. The micro expression training was not based on the Ekman et al.'s (Paul Ekman Group, 2020a) commercial training. Ekman's et al.'s training consists of several components including instruction, practice with feedback and review of presented material. However, research suggests that practice with feedback is the most effective component for training ERA and interpersonal accuracy (Blanch-Hartigan, 2012; Blanch-Hartigan et al., 2012; Blanch-Hartigan and Ruben, 2013), and that there was no association between length of training and effectiveness (Blanch-Hartigan et al., 2012; Rebeschini et al., 2019). On the contrary, shorter trainings are likely preferable in terms of saving resources and upholding motivation. The micro expression training in the current study consisted of training with feedback, using a basic emotion approach. An active control training consisted of an emotional working memory task. Outcome was measured with a multimodal ERA task, a micro expression ERA task and a third computerized measure about patients' emotional cues. Schlegel et al. (2017) reported transfer effects from the audio-visual training to unimodal tasks using different kinds of items. In the present study, transfer effects among all three outcome measures were investigated, with the idea that training one ERA facet could create increased awareness for emotional expressions and trigger inner processing of various kinds of socio-emotional stimuli. Especially the ecologically valid multimodal task should induce transfer effects. The exploration of transfer effects was the primary function of the third outcome measure about patients' emotional cues. Further, we were interested in how baseline ERA was related to improvements in ERA, suggesting that low baseline scores might leave more room for improvement, and in exploring training trajectories to understand whether all training sessions were necessary to train ERA (participants trained once weekly for three consecutive weeks).

### Hypotheses and Exploration

1. We hypothesized that there would be a significantly higher increase in ERA for the ERA training groups, as measured by their respective outcome measure, compared to the other two groups. In other words, the multimodal training group should show a significantly higher improvement in recognizing emotional expressions in multiple modalities than the micro expression training and control training groups; whereas the micro expression training group should show significantly higher improvement in the micro expression measure than the multimodal and control training groups.
2. For multimodal ERA, we explored training effects separately for each presentation modality (audio, video, audio-video), for positive and negative emotions, and for high and low arousal emotions.
3. We hypothesized that there would be small transfer effects (i.e., effects of the multimodal training on micro expression recognition and patient emotion cue recognition; effects of micro expression training on multimodal ERA and patient emotion cue recognition).
4. We explored how baseline ERA was related to improvements in ERA.
5. We explored the training trajectories of the two training groups.

## Materials and Methods

### Participants and Recruitment

Seventy-two healthy undergraduate students enrolled in the study (*M* = 24.69, *SD* = 7.69, range = 18–51 years, 54 women), of which 67 completed both measurements and all three training sessions (*M* = 24.45, *SD* = 7.38, range = 18–51 years, 49 women). Three participants dropped out after the pretest and did not start with the training at all. One student terminated participation after the second training session, and one after the third training session. We do not have information as to why the terminations occurred. Analyses and descriptive statistics include only complete cases.

All of the participants attended some undergraduate psychology class at Stockholm University (basic level) and participated for course credits. Recruitment was conducted via posting boards at the university and email lists for students that needed to participate in research. Apart from one student, none had any form of clinical training or experience, and none had participated in a socio-emotional training program before. The participants were randomized to one of the training programs or the active control condition. Previous research shows that women generally are slightly better at emotion recognition than men (e.g., Thompson and Voyer, 2014; Hall et al., 2016a), thus, we stratified for gender. There were no significant age differences between the groups, χ^2^(2) = 0.56, *p* = 0.79, according to a Kruskal Wallis rank sum test. Twenty-one people participated in the multimodal training, 23 in the micro expression training and 23 in the control training.

### Procedure

In the first lab session, participants completed a set of self-assessment questionnaires (explicit and implicit affective state, sleepiness, subjective ERA, empathy, adult attachment; see Supplementary Material for information on the measures, results and discussion of the results). Then, they completed three computerized ERA tasks to determine a baseline level of their ERA (detailed below). One week after the pretest, the three-week training phase started. Participants trained once a week (ca. 15 min per session) at the lab facilities, when possible with the same time intervals in-between sessions. We decided for a three-week training phase to give enough time for consolidation of emotion recognition skills. In the first training session, the two experimental groups also watched a short video lecture about emotional expressions (ca. 10 min). The participants were blind to their condition. The test leaders were present during the training sessions to support the participants with practical matters; thus, the study design was only single-blind. The tests and trainings were conducted individually, however, for practical reasons multiple participants could train at the same time. The posttest took place approximately 1 week after the last training session and consisted of the ERA tasks, as well as questions about affective state, sleepiness, and subjective ERA (see Supplementary Material) and a short qualitative evaluation of the training programs. In the end, participants were debriefed.

### Emotion Recognition Accuracy Tasks

The *Emotion Recognition Assessment in Multiple Modalities* test (ERAM; Laukka et al., 2021) is a computerized task to assess different modalities of nonverbal ERA. The 72-item task uses items from the GEMEP corpus (Bänziger et al., 2012)—a set of dynamic video clips of emotional expressions—and is divided into three blocks using different kinds of items: video, audio, or audio-video presentations of nonverbal emotional expressions. By this, the test provides separate measures for these three modalities. In the video modality, the participant needs to infer which emotion an actor is expressing based on their facial expression and body language. In the audio modality, the participant needs to infer the emotion based only on prosody without any visual information. To exclude the influence of verbal content, the actor expresses the emotion in a pseudo- language (“ne kal i bam sud molen” and “kun se mina lod belam”). In the audio-video modality, the participant is provided both visual and auditory information. The GEMEP clips include five female and five male professional actors of different ages that recorded the emotional expressions under the guidance of a professional theater director (see Bänziger et al., 2012). The emotions used were *anger, anxiety, despair, disgust, fear, interest, joy, pleasure, pride, relief* , *irritation*, and *sadness*. They vary in their valence and degree of arousal. Each modality (consisting of 24 items) depicts two displays of each of these 12 emotions. The task consists of identifying as fast as possible which emotion was displayed and to match it with a predefined list of emotions using the computer mouse. The task was conducted in Swedish. The internal consistency of the test in the current sample was α_pre_ = 0.67 and α_post_ = 0.66, using Kuder-Richardson Formula 20 for dichotomous data (KR-20; Kuder and Richardson, 1937); whereas in an evaluation study (Laukka et al., 2021), the ERAM showed stronger reliability.

As a measure of recognition accuracy for micro expressions, we developed a 70-item computer task (*micro expression recognition task*; MICRO) using colored still pictures from the Radboud Faces Database (Langner et al., 2010) depicting both female and male young faces. A frontal picture of an actor's face displaying an emotional expression is presented for 200 ms and is both preceded and followed by a neutral expression that lasts for 2 s. Using this double-masking technique, a micro expression is created from still pictures. The actors were trained to express facial emotional expressions according to prototypes from the *Facial Action Coding System* (Ekman et al., 2002). The 70 items for each measurement were randomly selected from a pool of 312 items. The emotions used were seven of the basic emotions proposed by Ekman (e.g., Ekman and Cordaro, 2011): *happiness, surprise, fear, disgust, sadness, anger, contempt*. The participants were instructed to indicate which micro expression they perceived based on a list of emotions, using the keyboard (forced choice format). They were instructed to answer as fast and as accurately as possible; the task was conducted in Swedish. In the current sample, the KR-20 internal consistency was α_pre_ = 0.84 and α_post_ = 0.86.

A slightly modified and computerized version of the *Patient Emotion Cue Test* (PECT; Blanch-Hartigan, 2011) was used to assess the accuracy for detecting verbal and nonverbal emotional displays that are representative of the medical clinical context. In 47 video clips, a young female actor is displaying the five emotions *anger, sadness, happiness, anxiety*, and *confusion*, as well as neutral expressions, via a combination of nonverbal (prosody, face, body language) and verbal (content) information. The actor plays the role of a patient that is talking to a medical professional. The emotional statements that she is conveying were derived from real patient interactions (e.g., “It's just being gradually getting worse” or “Can I play golf again?”) and the verbal and nonverbal expressions vary in their intensity. The clips were averaging 3 s and were followed by a black screen (8 s) as response window. The PECT has been found to be a reliable and valid tool for assessing patients' emotional cues in the medical context (Blanch-Hartigan, 2011). In the current sample, the KS-20 internal consistency was α_pre_ = 0.61 and α_post_ = 0.57.

### Emotion Recognition Training Programs and Control Training

In the multimodal ERA training, the participants were trained in the ability to recognize nonverbal emotional expressions in different modalities (audio, video, audio-video). The training was based on the multimodal approach of the ERAM (Laukka et al., 2021), using items from the extended GEMEP corpus (Bänziger et al., 2012) that were not used in the ERAM to avoid recollection effects. Each session, the program randomly chose 2 items per emotional expression per modality out of a pool of 144 items (each training session consisted of 72 items). First, the easiest condition consisting of audio-video items was conducted, followed by the video-only and audio-only conditions. Same as in the ERAM, after watching an emotional expression, the participants chose their answer from a list of emotions. Then they received immediate feedback on whether their answer had been correct, and, if not, about the correct answer. In the end of each training session, they received extended feedback about their typical errors by means of an individual confusion matrix. In the first of the three training-sessions, the participants also watched an informative 15-min video lecture about emotions and emotional expressions.

The micro expression ERA training followed the same principle as the MICRO. The participants viewed an emotional facial expression that was double-masked with a neutral expression and were asked to indicate which of the suggested emotions had been presented. The same emotion categories were used as for the micro expression task, with the exception of anger. Due to a coding error, anger was not included in the training. The change in anger recognition will be reported separately in the *Results* section. The items were derived from a different database, the *Karolinska Directed Emotional Faces* dataset (Lundqvist et al., 1998). For each session, 60 items that were randomly selected from a pool of 336 items were used to train micro expression ERA. After each answer, the participants received immediate feedback about the accuracy of their answer, as well as the correct answer in case of misattribution. At the end of the training, the participants further received their individual recognition rates per emotion. In the first of the sessions, the participants in this training group also watched the video lecture about emotions and emotional expressions.

The active control training consisted of an emotional working memory task with *N*-back format (see Gerhardsson et al., 2019) that also included some immediate feedback in the beginning of the task. Different pictures with positive, negative and neutral valence taken from the *International Affective Picture System* (Lang et al., 2008) were presented and participants had to decide whether a certain picture had already been displayed in the previous presentation (block 1 and 2), or three presentations before (block 3 and 4). Each of the four blocks consisted of 72 items. After the training phase of the task, the participants neither received further feedback about their performance nor any extended feedback in the end. They did also not watch the informative video lecture. The task was chosen because it was deemed to be closely enough related to the idea of recognizing emotions, but presumably unrelated to emotion recognition capacities when it comes to human displays of emotion. The presence of training aspects in the beginning of the task should further “mask” the control condition.

### Design, Data Preparation, and Analyses

The present study is a randomized controlled, single-blind study investigating the effectiveness of two ERA training programs. *R* (R Core Team, 2020, v. 3.6.3) and *RStudio* (RStudio Team, 2020, v. 1.1.456) were used for data preparation and data analyses. In accordance with standards in the field and to prevent bias due to false alarms, we used Wagner's (1993) unbiased hit rate (H_u_) for the ERA scores prior to statistical analyses. The H_u_ is a way of controlling for response bias, namely for how often an emotion category was used incorrectly (and not only the average number of correct answers for a certain emotion). Taking into account false alarms has been shown to be relevant in ERA training studies (see e.g., Griffiths et al., 2015). Arcsine transformation of H_u_ data is generally recommended (Wagner, 1993), though criticized by some researchers (e.g., Warton and Hui, 2011). The present data did not profit from arcsine transformation or other transformations for normalization, which is why we decided against it. Instead, we used non-parametric alternatives whenever necessary. We also decided against exclusion of outliers, since the outliers displayed high ERA scores that likely represent true values. The questionnaire data showed several violations against the assumption of normality. Also here, we decided against transformation and capping of outliers and used non-parametric alternatives when needed. We applied a 5% alpha level for significance tests, however, for transparency we also report exact *p*-values.

The ERAM outcome can be divided into modalities and according to valence and arousal categories. However, as primary outcome we used the ERAM total score, the mean H_u_ across all modalities and categories. For looking into the data according to valence, we calculated separate scores for positive and negative items of the ERAM. Positive items were relief, interest, pleasure, joy, and pride; negative items were irritation, anxiety, sadness, anger, fear, despair, and disgust. We also looked at high and low arousal items separately. High arousal items were joy, pride, anger, fear, and despair; low arousal items were relief, interest, pleasure, irritation, anxiety, and sadness. The authors of the GEMEP list disgust as an emotion without specified arousal level (see Bänziger et al., 2012). For the valence and arousal categories we used the ERAM total scores and did not differentiate further according to modality. We did not divide the MICRO and the PECT into positive and negative items since there was only one clear positive expression (happiness), and not according to arousal.

Because assumptions for parametric Analysis of Variance (ANOVA) were violated in some ERAM conditions, the Aligned Rank Transform (ART; Wobbrock et al., 2011) for mixed factorial designs was used as a non-parametric alternative to answer our main research question (effectiveness of trainings). We applied a 2 × 3 mixed factorial design. The within factor was time (pre, post), the between subjects' factor was training (multimodal, micro expression, control). We also included a random intercept with participant as grouping variable. ART is a modification of the Rank Transform (Conover and Iman, 1981) that allows for accurately testing for interaction effects. By aligning the data to strip the interaction effect from the main effects (as well as the main effects from each other and the interaction) and then ranking it, a mixed factorial ANOVA is made possible (R package *ARTool*, Kay and Wobbrock, 2020). We followed up with *post-hoc* ART contrast analyses using the R package *phia* (De Rosario-Martinez, 2015) that allow for testing the difference between differences for the interaction, meaning that we were looking at whether the pre–post difference for one training group was significantly different from the pre–post difference of another group (pairwise, Holm adjusted). For consistency reasons, the ART analyses were used for all ERA measures. Standardized effect sizes were not computable due to unavailability of Type III sum of squares for mixed models. Instead, we calculated Cohen's *d*_*z*_ scores for the pre–post differences for each group separately to compare the effects between groups. Cohen's *d*_*z*_ is the effect size for the standardized mean difference for within-subjects designs, based on the standard deviation of the difference (Lakens, 2013) and is interpreted like Cohen's *d* (traditionally *d* = 0.02 as small; *d* = 0.05 as medium; *d* = 0.08 as large; see Cohen, 1988). Violin plots including box plots visualize the group differences from pre to post for the three outcome measures (R package *ggplot2*, Wickham, 2016).

For exploring the association between baseline ERA and improvements in ERA, we conducted Spearman correlation analyses between baseline ERA and the pre–post improvement for the ERAM total score and the MICRO. For the analysis of the training trajectories and for descriptive purposes, we used parametric one-way ANOVA, or Kruskal-Wallis one-way ANOVA of ranks in instances of non-parametric data, to explore differences between the training groups. For estimating the magnitude of an effect, we applied following common interpretations: η^2^ = 0.01 (small), η^2^ = 0.06 (moderate), η^2^ = 0.14 (large); and, ε^2^ = 0.01 (small), ε^2^ = 0.08 (moderate), ε^2^ = 0.26 (large). Unpaired Wilcoxon signed rank tests (Holm adjusted *p*-values) were used for non-parametric *post-hoc* analyses of group differences. For the micro expression training group, the absence of anger items made it impossible to calculate H_u_ scores for the three training sessions. The category *anger* was used 24 times in session 1, 13 times in session 2, and 10 times in session 3, although no angry faces were displayed, leading to a discrepancy between frequency used and frequency correct emotion categories. For that reason, we used the frequency correct (H) scores for analysis of the training trajectory in the micro expression training group.

Paired Wilcoxon signed rank tests were used for investigating differences between pre and postscores. Gender differences in ERA were assessed using one-sided student's *t*-tests or independent 2-group Mann Whitney U tests. For analysis of internal consistency of the measures, we used the R package *DescTools* (Signorell, 2021) and the GitHub R package *validateR* (Desjardins, 2015). Additionally to our hypotheses and research questions, we explored associations between ERA and relevant trait variables (empathy, adult attachment) and subjective ERA, and investigated possible influences of affective state and sleepiness on ERA (see Supplementary Material).

### Statistical Power

An a priori power analysis for repeated measures within-between interaction ANOVA was conducted using the program G^*^Power (v. 3.1.9.3; Faul et al., 2009). Aiming for a power of 90% and predicting a medium effect (*f* = 0.25) for the training groups in their respective outcome measure and a pre–post correlation of *r* = 0.5 (3 groups, 2 measurements, α error probability = 0.05), a sample size of 54 was advised. Still, we decided for a slightly larger sample anticipating dropouts and for comparability with another sample (see https://osf.io/3y2gb/). A power analysis based on non-parametric ANOVA was not available. The use of non-parametric statistical analyses can often impair power; not much is published on the statistical power of ART. However, Leys and Schumann (2010) report results of a Monte Carlo simulation that showed that in cases of deviations from the normality assumption and deviations from normality and heteroscedasticity assumptions, the ART was a more powerful tool than parametric ANOVA. The difference in statistical power increased linearly with magnitude of deviation. Following this, the statistical power for the analyses investigating the hypotheses of the preset study can be interpreted as good. The exploratory analyses need to be interpreted with caution.

### Preregistration and Ethical Considerations

The present study was part of a research project about training ERA in the psychotherapy education and was approved by the regional ethical review board in Stockholm, Sweden (dnr 2015/1948-1931). All participants signed an informed consent form prior to participation. The present study is an extension of a study that investigates trainee psychotherapists' ERA and how it can be trained in the clinical psychology education and data collections have been conducted concurrently. Study design, methods, sample size and research questions were published on Open Science Framework (https://osf.io/3y2gb/). There are no known negative side effects of ERA trainings, although misattributions of emotions or subjective problems in recognizing emotional expressions could potentially lead to frustration for participants. To counter this, test leaders were available to the participants at all times. Since the control group did not receive an ERA training, they were offered the opportunity to take part in the real training after debriefing, though none accepted this offer.

## Results

Table 1 depicts H_u_ scores for overall ERA in the three ERA tasks—and the different modality, valence, and arousal conditions for the ERAM test—pre- and post-intervention for the three groups, as well as group comparisons and effect sizes. There were no significant differences in any of the ERA scores between the groups in the pretest, suggesting that the randomization created equal groups. The significant group differences in the posttest are explored in the ART ANOVAs below. There were no gender differences in any of the ERA variables at any time point (according to one-sided student's *t*-tests and independent 2-group Mann Whitney *U*-tests). In Supplementary Table 1, the reader can find descriptive statistics for the single emotions of the three ERA measures (pre/post) per training group.

**Table 1:** Descriptive statistics (means, standard deviations, 95% confidence intervals) and comparisons of the three groups for the ERA test variables.

**Measures**	**Multimodal training**	**Micro expression training**	**Control training**	**Overall**	***F*/χ^2^**	**η^2^/ε^2^**
ERAM total (pre)	0.35 (0.09) [0.32, 0.40]	0.36 (0.10) [0.32, 0.40]	0.41 (0.10) [0.37, 0.45]	0.38 (0.10) [0.35, 0.40]	χ^2^(2) = 4.53 (p = 0.10)	ε^2^ = 0.07
ERAM total (post)	0.51 (0.10) [0.47, 0.55]	0.42 (0.09) [0.38, 0.46]	0.47 (0.10) [0.42, 0.51]	0.47 (0.10) [0.44, 0.49]	*F*_(2, 64)_ = 4.08 (*p =* 0.02)^*^	η^2^ = 0.11
ERAM audio (pre)	0.33 (0.09) [0.29, 0.37]	0.35 (0.13) [0.30, 0.41]	0.38 (0.13) [0.33, 44]	0.36 (0.12) [0.33, 0.39]	*F*_(2, 64)_ = 1.17 (*p =* 0.32)	η^2^ = 0.04
ERAM audio (post)	0.48 (0.13) [0.43, 0.54]	0.41 (0.12) [0.36, 0.46]	0.43 (0.13) [0.37, 0.48]	0.44 (0.12) [0.41, 0.47]	*F*_(2, 64)_ = 2.16 (*p =* 0.12)	η^2^ = 0.06
ERAM video (pre)	0.42 (0.12) [0.37, 0.48]	0.38 (0.11) [0.33, 0.43]	0.44 (0.11) [0.39, 0.49]	0.41 (0.12) [0.38, 0.44]	*F*_(2, 64)_ = 1.57 (*p =* 0.22)	η^2^ = 0.05
ERAM video (post)	0.56 (0.14) [0.51, 0.65]	0.46 (0.13) [0.41, 0.52]	0.50 (0.12) [0.45, 0.55]	0.51 (0.13) [0.47, 0.54]	*F*_(2, 64)_ = 3.42 (*p =* 0.04)^*^	η^2^ = 0.10
ERAM audio-video (pre)	0.48 (0.13) [0.42, 0.54]	0.52 (0.17) [0.44, 0.59]	0.58 (0.13) [0.53, 0.64]	0.53 (0.15) [0.49, 0.56]	*F*_(2, 64)_ = 2.68 (*p =* 0.08)	η^2^ = 0.08
ERAM audio-video (post)	0.60 (0.11) [0.55, 0.65]	0.54 (0.14) [0.48, 0.61]	0.62 (0.12) [0.57, 0.68]	0.59 (0.13) [0.56, 0.62]	*F*_(2, 64)_ = 2.34 (*p =* 0.11)	η^2^ = 0.07
ERAM positive valence (pre)	0.40 (0.11) [0.35, 0.45]	0.39 (0.12) [0.34, 0.44]	0.43 (0.11) [0.39, 0.48]	0.41 (0.11) [0.38, 0.43]	*F*_(2, 64)_ = 0.90 (*p =* 0.41)	η^2^ = 0.03
ERAM positive valence (post)	0.58 (0.13) [0.52, 0.63]	0.48 (0.13) [0.43, 0.54]	0.50 (0.09) [0.46, 0.54]	0.52 (0.12) [0.49, 0.55]	*F*_(2, 64)_ = 4.01 (*p =* 0.02)^*^	η^2^ = 0.11
ERAM negative valence (pre)	0.33 (0.11) [0.28, 0.38]	0.34 (0.12) [0.29, 0.39]	0.40 (0.12) [0.35, 0.45]	0.36 (0.12) [0.33, 0.38]	*F*_(2, 64)_ = 2.42 (*p =* 0.09)	η^2^ = 0.07
ERAM negative valence (post)	0.46 (0.12) [0.41, 0.51]	0.38 (0.11) [0.34, 0.43]	0.45 (0.15) [0.38, 0.51]	0.43 (0.13) [0.40, 0.46]	*F*_(2, 64)_ = 2.39 (*p =* 0.10)	η^2^ = 0.07
ERAM high arousal (pre)	0.37 (0.10) [0.33, 0.42]	0.37 (0.11) [0.32, 41]	0.41 (0.10) [0.36, 0.45]	0.38 (0.10) [0.36, 0.41]	*F*_(2, 64)_ = 1.07 (*p =* 0.35)	η^2^ = 0.03
ERAM high arousal (post)	0.53 (0.13) [0.47, 0.58]	0.44 (0.16) [0.39, 0.49]	0.48 (0.10) [0.44, 0.52]	0.48 (0.12) [0.45, 0.51]	*F*_(2, 64)_ = 3.37 (*p =* 0.04)^*^	η^2^ = 0.10
ERAM low arousal (pre)	0.55 (0.12) [0.50, 0.61]	0.55 (0.08) [0.52, 0.59]	0.60 (0.11) [0.55, 0.65]	0.57 (0.11) [0.54, 0.59]	*F*_(2, 64)_ = 1.74 (*p =* 0.18)	η^2^ = 0.05
ERAM low arousal (post)	0.66 (0.11) [0.61, 0.70]	0.61 (0.10) [0.57, 0.65]	0.65 (0.11) [0.60, 0.70]	0.64 (0.11) [0.61, 0.66]	*F*_(2, 64)_ = 1.43 (*p =* 0.25)	η^2^ = 0.04
MICRO (pre)	0.54 (0.14) [0.48, 0.60]	0.49 (0.14) [0.43, 0.55]	0.50 (0.17) [0.43, 0.57]	0.51 (0.15) [0.47, 0.54]	*F*_(2, 64)_ = 0.87 (*p =* 0.43)	η^2^ = 0.03
MICRO (post)	0.61 (0.14) [0.55, 0.66]	0.75 (0.15) [0.70, 0.81]	0.62 (0.11) [0.58, 0.66]	0.66 (0.14) [0.63, 0.70]	*F*_(2, 64)_ = 8.68 (*p =* 0.00)^***^	η^2^ = 0.21
PECT (pre)	0.41 (0.11) [0.36, 0.46]	0.39 (0.12) [0.34, 0.45]	0.44 (0.08) [0.40, 0.48]	0.41 (0.11) [0.39, 0.44]	*F*(2, 63) = 1.22 (*p =* 0.30)	η^2^ = 0.04
PECT (post)	0.47 (0.12) [0.41, 0.52]	0.45 (0.08) [0.41, 0.48]	0.48 (0.13) [0.43, 0.54]	0.46 (0.11) [0.44, 0.49]	*F*(2, 63) = 0.61 (*p =* 0.55)	η^2^ = 0.02

*N = 67. PECT data for one participant (multimodal training group) lost due to technical reasons. The ERA scores (range 0–1) are presented as unbiased hitrates (H_u_)*.

* *p < 0.05*,

*** *p < 0.001*.

### ERAM

The ART ANOVA revealed that there was no main effect of training group, *F*_(2, 64)_ = 2.28, *p* = 0.11, on the ERAM total score (primary outcome measure for the multimodal training), but that the main effect of time was significant, *F*_(2, 64)_ = 76.21, *p* < 0.000. A Wilcoxon signed rank test with continuity correction revealed that the median posttest ERA was significantly higher than that of the pretest irrespective of training group (*V* = 194, *p* < 0.00), with a mean increase of 0.09 points (9%). More importantly, the interaction between time and training group was significant, *F*_(2, 64)_ = 6.83, *p* < 0.002 (see Table 2).

**Table 2:** Non-parametric mixed factors ANOVA (time × training) using the Aligned Rank Transform.

**Predictor**	***N***	***df***	***F***	***p***
ERAM total	67			
Training		2	2.28	0.11
Time		1	76.21	0.000^***^
Training × Time		2	6.83	0.002^**^
ERAM audio	67			
Training		2	0.47	0.63
Time		1	28.07	0.000^***^
Training × Time		2	4.47	0.02^*^
ERAM video	67			
Training		2	2.83	0.07
Time		1	29.77	0.000^***^
Training × Time		2	2.11	0.13
ERAM audio-video	67			
Training		2	2.58	0.08
Time		1	12.33	0.001^***^
Training × Time		2	2.62	0.08
ERAM positive valence	67			
Training		2	1.77	0.18
Time		1	62.43	0.000^***^
Training × Time		2	3.89	0.03^*^
ERAM negative valence	67			
Training		2	1.80	0.17
Time		1	33.78	0.000^***^
Training × Time		2	4.18	0.02^*^
ERAM high arousal	67			
Training		2	1.79	0.18
Time		1	47.87	0.000^***^
Training × Time		2	2.72	0.07
ERAM low arousal	67			
Training		2	1.35	0.27
Time		1	30.58	0.000^***^
Training × Time		2	2.35	0.10
MICRO	67			
Training		2	1.88	0.16
Time		1	57.47	0.000^***^
Training × Time		2	10.18	0.000^***^
PECT	66			
Training		2	1.28	0.28
Time		1	17.24	0.000^***^
Training × Time		2	0.50	0.61

*N = 67. PECT data for one participant (multimodal training group) lost due to technical reasons. Type III sum of squares*.

* *p < 0.05*,

** *p < 0.01*,

*** *p < 0.001*.

**Figure 1:**
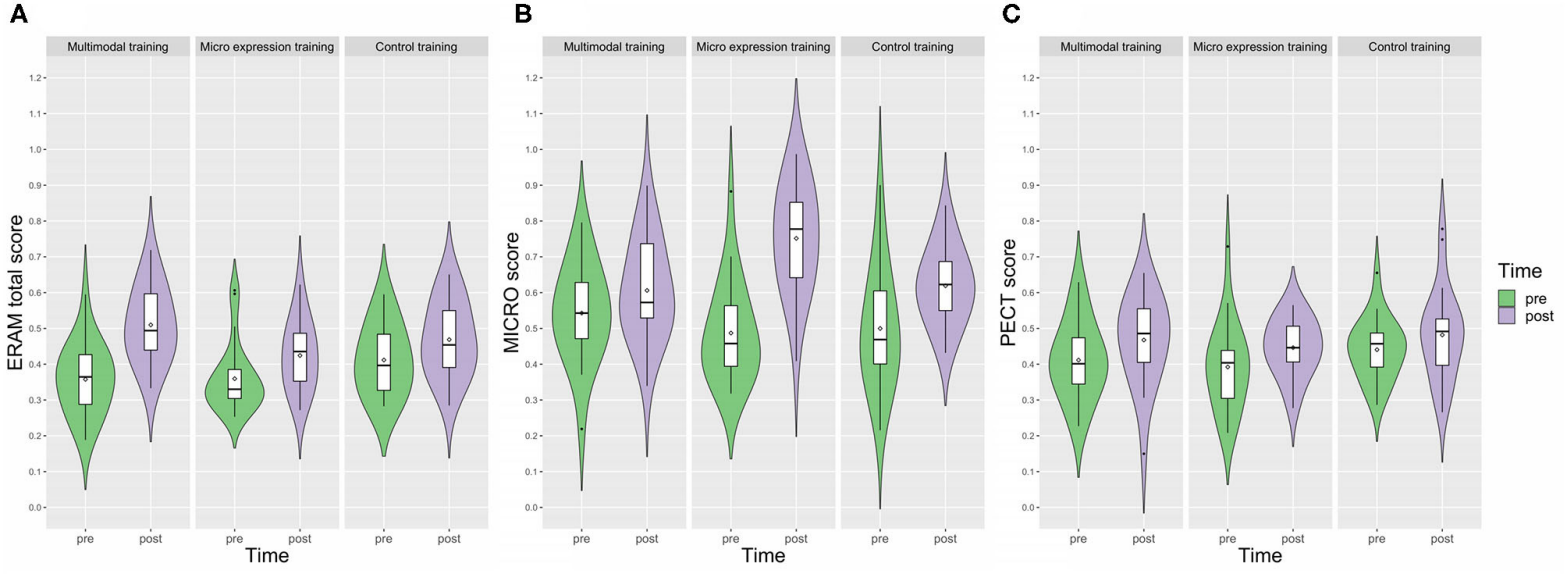
Violin plot with box plots of the training effects for the three main ERA measure: **(A)** ERAM total score, **(B)** MICRO score, **(C)** PECT score. The violin plots display the kernel probability density of the data at the different values for the three training groups. The box plots within the violin plots include the median (–) and the interquartile range (box), as well as the minimum and maximum (whiskers). Means were added in form of rhombuses. Small black dots display outliers.

We used ART interaction *post-hoc* contrast analyses (pairwise comparison, Holm adjusted, while subtracting out main effects; see Table 3) to answer the question whether the pre–post difference for the multimodal training group was significantly different from the pre–post differences for the other trainings, as would be expected according to our hypothesis. Although all participants became more accurate at detecting emotional expressions as assessed via the ERAM total score, *post-hoc* ART contrast analyses showed that the pre–post difference of the multimodal training group (diff = 0.15 points, i.e., 15%) was significantly higher than the pre–post difference of the micro expression training group (diff = 0.06 points, i.e., 6%), χ^2^ (1, *N* = 44) = 9.06, *p* = 0.005; and significantly higher than the pre–post difference of the CT group (diff = 0.06 points, i.e., 6%), χ^2^ (1, *N* = 44) = 11.57, *p* = 0.002. There was no difference of improvement between the micro expression training and the control training, χ^2^ (1, *N* = 46) = 0.16, *p* = 0.69. Figure 1A visualizes the ERAM total pre–post changes for the three training groups. The interquartile ranges of the pre–post scores for the multimodal training group do not overlap, which can be interpreted as evidence for a relevant difference of pre–post scores. In contrast, the pre–post interquartile ranges for the micro expression training group and for the control training group do overlap. The pre–post difference for the multimodal training group had a very large effect size (*d*_*z*_ = 2.04), whereas the micro expression training and control training groups displayed medium effect sizes (*d*_*z*_ = 0.64; *d*_*z*_ = 0.71; see Table 4). Altogether, this confirms the main hypothesis of this study regarding efficacy of the multimodal training.

**Table 3:** *Post-hoc* ART contrast analyses testing for significant differences between pre–post differences for the interaction effects (pairwise, Holm adj.).

**Interaction contrasts**	***n***	***df***	**χ^2^**	***p***
ERAM total				
1–2	44	1	9.06	0.005^**^
1–3	44	1	11.57	0.002^**^
2–3	46	1	0.16	0.69
ERAM audio				
1–2	44	1	5.23	0.04^*^
1–3	44	1	8.05	0.01^**^
2–3	46	1	0.32	0.57
ERAM video				
1–2	44	1	1.94	0.33
1–3	44	1	4.07	0.13
2–3	46	1	0.41	0.52
ERAM audio-video				
1–2	44	1	4.95	0.08
1–3	44	1	2.65	0.21
2–3	46	1	0.37	0.54
ERAM positive valence				
1–2	44	1	3.80	0.10
1–3	44	1	7.39	0.02^*^
2–3	46	1	0.62	0.43
ERAM negative valence				
1–2	44	1	5.81	0.03^*^
1–3	44	1	6.87	0.03^*^
2–3	46	1	0.05	0.83
ERAM high arousal				
1–2	44	1	4.08	0.12
1–3	44	1	4.20	0.12
2–3	46	1	0.00	0.98
ERAM low arousal				
1–2	44	1	3.47	0.16
1–3	44	1	3.69	0.16
2–3	46	1	0.00	0.95
MICRO				
1–2	44	1	19.50	0.000^***^
1–3	44	1	2.31	0.13
2–3	46	1	8.78	0.01^**^
PECT				
1–2	43	1	0.03	1
1–3	43	1	0.86	1
2–3	46	1	0.62	1

*N = 67. PECT data for one participant (multimodal training group) lost due to technical reasons. 1 = multimodal training, 2 = micro expression training, 3 = control training*.

* *p < 0.05*,

** *p < 0.01*,

*** *p < 0.001*.

**Table 4:** Mean pre–post differences for each group and ERA measure and pre–post effect sizes (Cohen's *d*_*z*_).

**Measure**	***n***	**diff*_***pre*−*post***_***	***d_***z***_***
ERAM total			
Multimodal training Micro expression training Control training	21 23 23	0.15 0.06 0.06	2.04 0.64 0.71
* ERAM audio*			
Multimodal training Micro expression training Control training	21 23 23	0.16 0.06 0.04	1.39 0.41 0.30
* ERAM video*			
Multimodal training Micro expression training Control training	21 23 23	0.14 0.08 0.07	1.23 0.58 0.54
* ERAM audio-video*			
Multimodal training Micro expression training Control training	21 23 23	0.12 0.03 0.04	0.96 0.17 0.43
* ERAM positive valence*			
Multimodal training Micro expression training Control training	21 23 23	0.18 0.09 0.07	1.79 0.74 0.67
* ERAM negative valence*			
Multimodal training Micro expression training Control training	21 23 23	0.13 0.05 0.05	1.41 0.37 0.47
* ERAM high arousal*			
Multimodal training Micro expression training Control training	21 23 23	0.16 0.07 0.07	1.23 0.54 0.85
* ERAM low arousal*			
Multimodal training Micro expression training Control training	21 23 23	0.10 0.06 0.05	1.28 0.59 0.44
MICRO			
Multimodal training Micro expression training Control training	21 23 23	0.06 0.26 0.12	0.45 1.33 0.75
PECT			
Multimodal training Micro expression training Control training	20 23 23	0.06 0.05 0.04	0.54 0.46 0.40

*N = 67. PECT data for one participant (multimodal training group) lost due to technical reasons. Cohen's d_z_ can be interpreted following Cohen's (1988) suggestion: d = 0.2 (small effect), d = 0.5 (moderate effect) and d = 0.8 (large effect)*.

Table 2 displays the results of the mixed factorial ART ANOVAs and Table 3 displays the *post-hoc* ART interaction contrasts; Table 4 displays the mean differences and pre–post Cohen's *d*_*z*_ scores per group for all ERA variables. The audio modality followed the same pattern as the ERAM total score (significant interaction contrasts for multimodal training vs. micro expression training and multimodal training vs. control training, but no significant difference of pre–post differences for micro expression training vs. control training), whereas the video only and audio-video modalities did not show interaction effects (see Tables 2, 3). When considering valence, there were interaction effects for both positive and negative valence items. For negative valence, the ERAM total and audio pattern was replicated (multimodal training vs. micro expression training and multimodal training vs. control training contrasts were significant); for the positive valence only the multimodal training vs. control training contrast was significant. Both arousal categories did not show interaction effects. In terms of standardized within-subjects effect sizes, the same pattern was established for all ERAM variables. The multimodal training showed large effects, whereas the micro expression training and control training displayed small to medium size pre–post effects (see Table 4).

### MICRO

For the MICRO (primary outcome for the micro expression training), we found both a main effect of time and a significant interaction (Table 2). In line with our main hypothesis regarding the micro expression training, the pre–post difference of the micro expression training group (diff = 0.26 points, i.e., 26%) was significantly higher than that of the multimodal training group [diff = 0.06 points, i.e., 6%; χ^2^ (1, *N* = 44) = 19.50, *p* < 0.001] and the control training group [diff = 0.12 points, i.e., 12%; χ^2^ (1, *N* = 46) = 8.78, *p* = 0.01], whereas there was no significant difference between the multimodal training and control training contrasts (Table 3). Figure 1B visualizes the pre–post changes of the three groups for the MICRO. The comparisons of the pre–post interquartile ranges support this result. The pre–post within subjects' effect was large for the micro expression training group and moderate or small for the multimodal training and control training group, respectively (Table 4).

Due to a coding error, the micro expression training did not include the emotion *anger*. For that reason, we performed a separate ART ANOVA for the anger items. Neither main effects, nor an interaction were found. Looking at the pre–post differences of the three groups descriptively, though (Supplementary Table 1), the micro expression training group displayed a large difference of 29%, compared to −3% (multimodal training) and 17% (control training).

### PECT

For the PECT outcome measure there was a main effect of time, but no interaction effect (Table 2). The pre–post differences of the three groups were small and comparable (4–6% improvement); standardized effect sizes were small to moderate for all three groups (Table 4). Figure 1C visualizes the pre–post changes of the three groups for the PECT; all interquartile ranges overlap.

### Transfer Effects

As reported above, the multimodal training group's ERAM pre–post change was significantly larger than that of the other groups and there was no difference in pre–post difference between the micro expression training and the control training groups. The equivalent was true for the micro expression training group and the MICRO results. Additionally, no differences between pre–post differences of the three groups were found for the PECT. Concluding from this, contrary to our hypothesis, no transfer effects of the two trainings could be detected.

### Association Between Baseline ERA and Improvement

Spearman correlation analyses showed a significant weak negative correlation between ERAM baseline score and pre–post improvement in multimodal ERA across all three groups, *r*_s_ = −0.37, *p* < 0.01, *N* = 67, though the association was not significant when considering the multimodal training group separately, *r*_s_ = −0.32, *p* = 0.16, *N* = 21. For the MICRO, there was a significant moderate negative correlation between baseline and pre–post improvement in micro expression ERA, both across the three groups, *r*_s_ = −0.65, *p* < 0.01, *N* = 67, as well as for the micro expression training group separately, *r*_s_ = −0.66, *p* < 0.01, *N* = 23. These results suggest that a low ERA baseline is associated with large improvement.

### Training Trajectories

Training trajectories for the multimodal training and micro expression training were analyzed using parametric and non-parametric one-way ANOVAs. An overview of the results and descriptive statistics of the sessions can be found in Table 5. Looking at the multimodal training group (*n* = 21), the group means rose steadily for all variables except for negative valence, however, the improvements were mostly small and/or did not reach significance. However, there were significant effects for training session number for the ERAM total score, ERAM video modality, negative emotions, and high arousal emotions; effect sizes were moderate. Group comparisons (Holm adjusted *p*-values) revealed the same pattern for ERAM total, video, and negative valence: The participants did not improve from session 1 to 2, but from session 1 to 3 and session 2 to 3. After correcting for multiple testing, no significant differences between sessions were found anymore for the high arousal emotions. There was no significant difference between the sessions for auditive ERA, audio-visual ERA, positive valence emotions, or low arousal emotions. In the micro expression training group (*n* = 23) there was a significant moderate to strong effect of training session. Pairwise comparisons using Wilcoxon rank sum test (Holm adjusted) revealed that there were significant differences between training session 1 and 2, as well as between 1 and 3; however, there was no significant improvement from session 2 to session 3.

**Table 5:** Descriptive statistics (means, standard deviations, 95% confidence intervals) and comparisons of the three training sessions for the ERAM and MICRO variables.

**Measures**	**Session 1**	**Session 2**	**Session 3**	***F*/χ^2^**	**η^2^/ε^2^**
Multimodal training group					
ERAM total	0.33 (0.07) [0.30, 0.36]	0.34 (0.09) [0.30, 0.39]	0.39 (0.09) [0.35, 0.43]	*F*_(2, 40)_ = 5.91 (*p =* 0.01)^**^	η^2^ = 0.09
ERAM audio	0.26 (0.10) [0.22, 0.31]	0.28 (0.11) [0.23, 0.33]	0.30 (0.13) [0.24, 0.36]	χ^2^(2) = 1.46 (*p =* 0.48)	ε^2^ = 0.02
ERAM video	0.40 (0.10) [0.36, 0.45]	0.41 (0.13) [0.35, 0.47]	0.48 (0.14) [0.42, 55]	*F*_(2, 40)_ = 5.24 (*p =* 0.01)^**^	η^2^ = 0.08
ERAM audio-video	0.48 (0.14) [0.42, 0.55]	0.49 (0.15) [0.42, 0.56]	0.55 (0.13) [0.49, 0.60]	*F*_(2, 40)_ = 1.58 (*p =* 0.22)	η^2^ = 0.04
ERAM positive valence	0.35 (0.08) [0.32, 0.39]	0.41 (0.13) [0.35, 0.47]	0.40 (0.11) [0.35, 0.45]	χ^2^(2) = 3.14 (*p =* 0.21)	ε^2^ = 0.05
ERAM negative valence	0.31 (0.10) [0.27, 0.36]	0.30 (0.10) [0.25, 0.34]	0.38 (0.09) [0.34, 0.42]	*F*_(2, 40)_ = 11.40 (*p =* 0.00)^***^	η^2^ = 0.12
ERAM high arousal	0.39 (0.09) [0.35, 0.43]	0.38 (0.09) [0.34, 0.42]	0.44 (0.11) [0.39, 0.49]	*F*_(2, 40)_ = 3.57 (*p =* 0.04)^*^	η^2^ = 0.07
ERAM low arousal	0.29 (0.08) [0.25, 0.33]	0.31 (0.11) [0.26, 0.36]	0.33 (0.08) [0.30, 0.37]	*F*_(2, 40)_ = 1.76 (*p =* 0.19)	η^2^ = 0.03
Micro expression training group					
MICRO	0.85 (0.07) [0.82, 0.88]	0.91 (0.07) [0.87, 0.94]	0.92 (0.08) [0.88, 0.95]	χ^2^(2) = 14.39 (*p =* 0.001)^***^	ε^2^ = 0.21

*n_multimodal training_ = 21; n_micro expression training_ = 23. Unbiased hitrates (H_u_) for ERAM variables, percentage correct (H) for the MICRO. Common effect size estimates: η^2^ = 0.01 (small), η^2^ = 0.06 (moderate), η^2^ = 0.14 (large); ε^2^ = 0.01 (small), ε^2^ = 0.08 (moderate), ε^2^ = 0.26 (large)*.

* *p < 0.05*,

** *p < 0.01*,

*** *p < 0.001*.

## Discussion

### Improvements Due to ERA Training

In sum, the results of the study show that the two training programs effectively improved ERA for their respective outcome measure and that the effects were substantial. Even though all three groups became better at recognizing nonverbal emotional displays in others (captured by all three ERA outcome measures), there were interaction effects for the ERAM total score and the MICRO. The pre–post improvement for the multimodal training group (in the ERAM outcome measure) was very large and significantly higher than for the micro expression training or the control training groups, while pre–post improvement did not differ significantly between the other two groups. An improvement of 15% compared to 6% in the other groups can be interpreted as relevant. The same pattern was observed for the micro expression training in the micro expression outcome measure. The micro expression training showed a large pre–post effect in the MICRO that was significantly higher than that of the multimodal training and control training groups. Again, there was no difference of improvement between the other two groups. In the micro expression training group, the difference in improvement was even more pronounced, showing a 26% improvement in micro expression recognition, compared to only 6% in the multimodal training group and 12% in the control training group, which can be called substantial. In the third outcome measure, the PECT, there was a general improvement of ERA, but no single group improved more than the others. This is not surprising, as no group trained in recognizing patients' emotional cues.

The results are in line with the hypotheses of the study. Each training significantly improved the participants' ERA for its respective facet—multimodal or micro expression recognition. The effects can be interpreted as substantial based on the contrasts (in percentages of improvement) and the standardized effect sizes for the pre–post change (Cohen's *d*_*z*_). A standardized effect size for the interaction effect such as η^2^ would have been desirable, but was not computable. Still, based on the unstandardized effect expressed in percentage of change and the standardized within-subjects effect sizes, the magnitude of the training effects can be interpreted as large.

The fact that all three groups improved in their ERA from pretest to posttest in all three measures was to be expected, as previous research shows that repeated exposure to an ERA task increases accuracy even without any training or feedback (see e.g., Bänziger et al., 2009). For all that, the large improvements for the experimental groups in relation to the other groups precludes explanation based solely on familiarity with items. The PECT results function as an additional control, showing that the observed training effects for multimodal ERA and micro expression ERA surpass repeated testing effects. Yet, it needs to be noted that using items from medical contexts makes the measure less relevant for a student sample and that the PECT employs both nonverbal and verbal dynamic expressions at the same time, which reduces its validity when it comes to nonverbal emotion recognition specifically.

It should be noted that the emotion *anger* was not trained in the micro expression training, due to a coding error. A separate analysis for the anger items suggested that there was no significant change in anger recognition measured by the MICRO in any of the three groups, though the inspection of the data showed a substantial (but non-significant) improvement in anger detection for the micro expression training group. This could be attributed to floor and ceiling effects which could have negatively influenced the ranking procedure, or to the small number of anger items. We note that ERA improvement can only be expected for emotions that are actually trained and that future versions of the micro expression training need to include anger.

The ERAM outcome measure provided the opportunity to explore the different modalities and the valence and arousal categories separately. Interestingly, the audio modality showed the same pattern as the ERAM total score. The multimodal training group had a significantly higher improvement in auditive ERA than both other groups, with comparable effect sizes. In the video and audio-video modalities, on the other hand, we could not find significant interaction effects or contrasts, even though standardized and unstandardized effect estimates followed the same pattern of large effects vs. small to moderate. It could be concluded that the improvement in auditory ERA is the driving force behind the ERA improvements of the multimodal training group, even though relatively higher improvements can be observed in all modalities. This could be due to the fact the multimodal training group was the only group that trained auditory ERA, which makes it not surprising that this group had the biggest improvement in the audio modality. The increased exposure to and training of auditory cues and possibly awareness for sending and receiving emotional content via the voice, was exclusive to these participants. However, it has to be noted that the lack of significant effects for the other modalities could also be due to the small number of items per modality. Following research that indicates that nonverbal emotional communication heavily relies on information beyond facial expressions (e.g., Bänziger et al., 2012; Rigoulot and Pell, 2012; Paulmann et al., 2013), like bodily postures, prosody or vocalizations, it is promising that it is possible to train auditory emotion recognition with a brief computerized program. To our knowledge, the present study is the first to systematically investigate a unimodal auditory ERA training component. Future research needs to replicate this finding and shed light on mechanisms underlying auditory emotion recognition learning.

In regards to valence of multimodal emotional expressions, there were significant interactions for both positive and negative emotions. For negative emotions, the same pattern as for ERAM total could be shown. The multimodal training group improved significantly more than the other two groups. The training was effective for the six negative valence emotions trained in the multimodal training and the differences in standardized and unstandardized effects were large. For positive emotions, there was a significant difference between the multimodal training and control training pre–post improvements. However, there was no difference between the multimodal training and the micro expression training group. The micro expression training, in addition to happiness, included the emotion *surprise*, which could be understood as a positive as well as a negative emotion. This result could tentatively be interpreted as a small transfer effect from the micro expression training to positive emotions of the ERAM. Still, the absence of an effect cannot be interpreted as evidence for a null effect and it remains unclear why there was no difference of improvement between the multimodal training and the micro expression training group for positive emotions. Also here, the small number of items could have played a role. Overall, it is intuitive that the multimodal training group would get better at detecting both negative and positive emotions in the ERAM, as the emotion categories trained were different from the micro expression training and control training. Interestingly, the improvements for positive emotions were generally larger than for negative emotions. When considering high and low arousal emotions, all groups improved and no significant differences in improvement could be found, even though the trends go in the same direction as for the other ERAM variables. Notably, the smallest ERA improvements were observed for the low arousal items that tend to be more difficult to detect.

### Transfer Effects

The study also wanted to explore the possibility of general improvements in ERA due to the trainings. For example, whether a participant trained in multimodal ERA would also become better at detecting micro expressions (or vice versa) because of an improvement of a general ability to recognize nonverbal emotions in others or possibly due to an increased awareness of nonverbal emotional expressions. However, this was not found to be the case. Even though we had hypothesized transfer effects between the two training groups, it is not counterintuitive that those did not occur. Even though Schlegel et al. (2017) found transfer effects in some of their studies, the transfer happened from related emotion facets. Their audio-visual training improved recognition of emotional faces or voices, respectively, in two samples. In the present study, the difference between the two ERA facets—multimodal ERA and micro expression ERA—likely was too big, meaning that the ability to read very brief facial emotions is too specific to be trained by learning to detect nonverbal auditory ERA or dynamic long lasting video stimuli that do not rely on attention for very fast shifts in facial expressions. The same logic applies vice versa. Learning to detect very fast shifts in facial emotional expressions does not prepare one to detect dynamic stimuli, especially not audio-only stimuli, the most unfamiliar and difficult expression modality. Furthermore, the stimuli used for the two trainings (videos/audio sequences vs. still pictures) might have been too different to induce transfer effects.

The PECT was included as a third, independent outcome measure in which none of the participants were trained as a further means to investigate transfer effects. The results from the PECT corroborate the results of the other two measures. All three groups improved significantly from pre- to posttest in the PECT, but no group was superior over the other (general effect of time or repeated testing). Also here, we see that we only can expect people to get better at detecting nonverbal emotions in others for those facets of ERA that they were trained in. Even though it would have theoretically been plausible that the micro expression training group would have improved slightly more than the other two groups in the PECT since it was based on similar emotion categories, this was not the case.

### Association Between Baseline ERA and Improvement

A low ERA baseline was associated with larger improvement in ERA from pre- to posttest. This was found to be a general tendency when considering all participants for both multimodal ERA and micro expression ERA. When exploring the association for the multimodal training group and the micro expression training group separately, the association was only significant for the micro expression training group, however, this could be due to the small sample size. It could be speculated that baseline ERA is relevant for pre–post improvement irrespective of whether a training is used or not. These results are not surprising, as there is less room for improvement for participants that are already good at ERA to begin with (ceiling effect). They are also encouraging, because ERA trainings are possibly more direly needed by individuals with low baseline ERA. The association was stronger for micro expression ERA than for multimodal ERA, which could be explained by the greater improvement in micro expression ERA compared to multimodal ERA and patient emotion cue ERA.

### Training Trajectories

The investigation of training trajectories uncovered interesting suggestions for future research. The participants generally increased their ERA from session to session, but those improvements were small and often not significant. For most of the multimodal ERA variables, no significant improvements between sessions could be detected, with the exception of the ERAM total score, the video modality, and negative emotions, where improvement seemed to occur in the later stages of the training schedule. It could be speculated that multimodal ERA training in itself and the use of a greater number of emotions are challenging and that participants need time to adjust to the training to profit from it. On the other hand do these results also sound counterintuitive, as the first session included an informative video lecture about emotional expressions, which could have “boosted” the increase between session 1 and 2. It needs to be noted that lack of statistical power (small subsample, use of non-parametric analyses, exploratory analysis) or the small number of items per modality and valence/arousal category could have contributed to the mixed findings. On a methodological note, it could have been relevant to include the pre- and post-scores in the training trajectory analysis as well. However, we decided against presenting this data, as there was a great dip in ERA from pretest to the training sessions, and a later strong increase from training session 3 to posttest. This finding is very likely due to methodological reasons, i.e., quality features of the used items, and not to real changes in ERA.

For training micro expressions of emotion, the opposite pattern was found. The micro expression training did not yield any significant improvements after training session 2. This suggests that future versions of the micro expression training could be sufficient including only two sessions, although further studies comparing versions of the trainings with varying numbers of sessions would be needed to answer this question. It can be noted that the micro expression ERA for all three training sessions was very high, much higher than for the pre and post assessments. Also in this instance, we assume that this was likely due to methodological reasons, such as easier items for the training sessions. The absence of anger expressions in the training makes it harder to generalize this finding.

### Strengths and Limitations

Comparing the training effects of the present study to those of other studies is difficult, as standardized effect sizes and experimental designs vary greatly among studies (Rebeschini et al., 2019). Yet, the present study has several advantages in regards to research design and methods, for example applying a randomized controlled mixed design that allows inferences of causality, increases statistical power and provides additional information about profiles of change. Further, we allowed for a week between last training session and posttest, accentuating the magnitude and external validity of the effects. In most previous studies the post measurement was done directly following the training. Previous studies often used the same items for training and outcome measurement, whereas the present study used different items for test and training. We can therefore exclude the possibility of effects being specific to learning certain items. The use of dynamic stimuli suggests that the findings of the multimodal training could be more generalizable than results of previous studies focusing on static pictures. The focus on multimodal ERA provided not only valuable insights about training ERA via different channels of nonverbal communication, but also insights about training auditive ERA unimodally. The micro expression training followed a double-masking procedure to create micro expressions. In future studies it would be relevant to investigate stimulus material containing naturally occurring micro expressions, as the dynamics of masked presentation of macro expressions likely could differ from leakage from concealed emotions (“true” micro expressions). The investigation of training trajectories could provide additional information about mechanisms behind ERA training, and the exploration of transfer effects could provide new insights about the specificity of ERA training results. A further advantage of the study was the use of emotion categories beyond basic emotions (especially the use of various positive emotions beyond *happiness*), a more ecologically valid approach to emotion recognition research, as emotions that are assumed to develop later in life and are largely learned and influenced by culture, should need more explicit training than emotions that are considered to be an evolutionary blueprint.

Several limitations need to be considered. One is that the study used a single blind design. The participants were not privy to whether they belonged to one of the experimental groups or the control group, but for practical reasons it was not possible to blind the two test leaders to these conditions. Moreover, the lack of counterbalancing of the ERA tasks in the lab could have led to fatigue and motivational declines from the MICRO to the ERAM to the PECT. Another methodological limitation is that, in case of the ERAM and the PECT, we used the same measure for pretest and the posttest. Nonetheless, using the same measure for pre and posttest is not uncommon in this line of research, and we hope to have alleviated recollection effects by allowing for approx. 4 weeks between pre and posttest. Also, recollection effects should at least theoretically influence all three groups equally. Using the exact same outcome measure can, on the other hand, be seen as an advantage, as it increases comparability and reduces the risk of measurement error due to item selection (e.g., degree of rater agreement, intensity of expression, video/audio quality, or other criteria). In fact, in the training trajectory analyses we decided to present only the three training sessions instead of all five timepoints, as there were decreases and increases in multimodal ERA and micro expression ERA that can only be explained by item selection. In the training trajectories for the three sessions, there was a clear upwards trend in all ERA variables, but the “baseline” was different from the outcome measurements. The MICRO, on the other hand, randomly selected its 70 items from a pool of 312 items, which ensured that the pre and the posttest were not identical. However, this also makes the pre–post comparison more difficult, as item difficulty was not controlled for. The reliability of the three ERA measures can also be discussed. Generally, an internal consistency of α > 0.7 is considered acceptable. This was only the case for the MICRO; the ERAM and the PECT showed values ranging from α = 0.57–0.67; whereas the ERAM showed greater reliability and the PECT lower reliability in evaluation studies (Blanch-Hartigan, 2011; Laukka et al., 2021). Blanch-Hartigan (2011) notes that lower internal consistency rates are not surprising in measures for nonverbal sensitivity that include relatively few items. Still, questionable or poor internal consistency of the ERAM and the PECT could hamper the validity of our results.

The use of our statistical methods could also be discussed. The decision against excluding outliers (even though convincing from a theoretical standpoint) can be criticized, as it introduced problems in regards to ANOVA assumptions and forced us to use non-parametric approaches. The ART ANOVA is an uncommon method in psychological science, which could limit the comparability with other studies. Further, the number of outcome measures and, following this, number of ANOVAs in this study can be criticized. However, hypotheses were only formulated for the two main outcome measures, and the ERAM modality, valence and arousal analyses were exploratory in nature. Other statistical approaches, such as multilevel mixed modeling could have been interesting alternatives, especially in regards to the training trajectory analyses. Nonetheless, we still see the use of this analysis as a good fit for our research questions and data.

## Conclusion and Future Directions

In closing, the present study shows that multimodal ERA and micro expressions ERA can be successfully trained using computerized training. Further, the present study is the first to train auditive ERA unimodally, which also led to improvements in auditive ERA specifically. The lack of transfer effects of the training suggests that ERA is a highly specialized skill and that ERA needs to be researched as a multifaceted construct. These results could also contribute to the debate about ERA as a unitary competence or set of related abilities (see e.g., Schlegel et al., 2012), which could be further explored in future studies. Future versions of the trainings could possibly include less sessions, making the self-administered training more feasible. The results are encouraging for healthy adults in general, as well as for specific populations that could profit from increased ERA, such as clinical populations, and professionals in the healthcare system, service sector or law enforcement.

Future efficiency studies are needed to investigate practical implications of ERA training (such as for mental health professionals and their patients), which is to date still a clear research gap. Also, the long-term stability of ERA training effects need to be researched, as well as the neural mechanisms and dynamics involved in ERA training. Finally, it needs to be understood that emotion recognition is embedded in dynamic interpersonal processes. Perceiving others' emotional expressions elicits own emotional responses that need to be processed, understood, interpreted, and regulated, before, if necessary, responding to the other. Subsequent socio-emotional processes, such as emotion regulation and empathy, as well as the importance of contextual factors have to be explored in tandem with ERA to be able to draw differentiated conclusions from ERA training research (see e.g., Bechtoldt et al., 2019). The complex processes involved in nonverbal socio-emotional communication via facial expressions, micro expressions, bodily postures, tone of voice, and other processes, such as biobehavioral synchrony (see e.g., Feldman, 2017, for a review), need to, eventually, be investigated as dynamics.

## Data Availability Statement

The raw data supporting the conclusions of this article will be made available by the authors, without undue reservation.

## Ethics Statement

The studies involving human participants were reviewed and approved by the regional ethical review board in Stockholm, Sweden (dnr 2015/1948-1931). The patients/participants provided their written informed consent to participate in this study.

## Author Contributions

LD, PL, LH, TB, IM, HF, and SH conceptualized the idea, participated in study planning, and writing of the manuscript. LD was primarily responsible for pre-registration, data collection, data analysis, and writing of the manuscript. PL, LH, and TB were responsible for the programming of the trainings and tests. TB is deceased. All authors contributed to the article and approved the submitted version.

## Conflict of Interest

The authors declare that the research was conducted in the absence of any commercial or financial relationships that could be construed as a potential conflict of interest.

## Publisher's Note

All claims expressed in this article are solely those of the authors and do not necessarily represent those of their affiliated organizations, or those of the publisher, the editors and the reviewers. Any product that may be evaluated in this article, or claim that may be made by its manufacturer, is not guaranteed or endorsed by the publisher.
