# Supplementary material for: Training Emotion Recognition Accuracy: Results for Multimodal Expressions and Facial Micro Expressions
Source: Front Psychol. 2021 Aug 12;12:708867. doi: 10.3389/fpsyg.2021.708867 (PMC8406528; doi:10.3389/fpsyg.2021.708867)
Supplement: Supplementary file 1 [file Data_Sheet_1.docx]

Supplementary Material

*Supplementary Table 1* displays descriptive statistics for single emotions of the ERA measures (pre/post) per training group. Additionally to our hypotheses and research questions, we explored associations between ERA and relevant trait and state variables. We produced a visualization of a correlation matrix (R package *corrplot*, Wei & Simko, 2017; see *Supplementary Figure 1*) to explore the relationships among the ERA variables and the state/trait variables (adult attachment, empathy, subjective multimodal ERA, subjective micro expression ERA, sleepiness, and explicit and implicit affective state). For investigating possible influences of affective state and sleepiness on ERA, we performed multiple regression analyses. Descriptive statistics, group comparisons and effect sizes for relevant trait and state variables can be found in *Supplementary Table 2*.

**Trait/state questionnaires (materials and methods)**

To be able to rule out the influence of sleepiness and affective state on ERA, we asked the participants to indicate their sleepiness and affectivity before starting the ERA tasks (pre- and posttest). Sleepiness was assessed via a Swedish version of the *Karolinska Sleepiness Scale* (KSS; Åkerstedt & Gillberg, 1990). The KSS is a one question measure in which the participants are asked how sleepy they felt during the preceding 5 minutes. The answering options span from 1 = *extremely alert* to 9 = *extremely sleepy, fighting sleep*.

A Swedish translation of the *Positive and Negative Affect Schedule* (PANAS; Watson et al., 1988) was used to (explicitly) assess positive and negative affectivity. It consists of ten positive emotional adjectives (e.g., enthusiastic, proud) as well as ten negative emotional adjectives (e.g., nervous, anxious), and the task to indicate on a 5-point Likert scale to which extent one experiences those emotional states at that moment (1 = *very slightly* to 5 = *very much*). In this sample, the positive affect scale had good internal consistency of α_pos_=.87 during both pre and posttest; and the negative affect scale had an acceptable reliability of α_neg_=.78 in the pretest and good reliability α_neg_=.86 in the posttest.

The *Implicit Positive and Negative Affect Test* (IPANAT; Quirin et al., 2009) is an indirect measure for positive and negative (trait and state) affectivity. Participants are asked to rate how much they think certain artificial words (e.g., SAFME) convey three positive and three negative moods (*happy*, *energetic*, *cheerful*, *helpless*, *tense*, *inhibited*). The rating scale stretches from 1 = *doesn’t fit all* to 4 = *fits very well*. The assumption behind this indirect test is that a person’s affectivity influences their evaluative processes. The way in which artificial words are experienced as positive or negative conveys information about participants’ state and trait affect, though subjective associations in response to the artificial words cannot be ruled out (Quirin et al., 2018). Research with the IPANAT shows good psychometric properties (see Quirin et al, 2009; Quirin et al., 2018), and in the current sample, the test had had good reliability in the pretest (α_pos_=.88; α_neg_=.83) and acceptable/good reliability in the posttest (α_pos_=.79; α_neg_=.84).

A self-designed questionnaire was used to assess the participants’ subjective perception of their ERA. Four questions were about estimating their multimodal ERA (based on different sources of information: facial expression, body language, prosody, overall). Four questions were specifically about estimating micro expression ERA. Internal consistency of the two subscales was calculated and found to be acceptable during both pre- and posttest (multimodal ERA: α_pre_ =. 77 and α_post_ = .75; micro expression ERA: α_pre_ = .8 and α_post_ = .78). However, it should be noted that Cronbach’s alpha is sensitive to small item number.

We used two different questionnaires to assess empathy. Both try to capture the two aspects of empathy: cognitive empathy and affective empathy. The *Interpersonal Reactivity Index* (IRI; Davis, 1983) is by many considered to be the gold standard for assessing empathy via self-report. It consists of 28 items for which the participant is asked to indicate on a 5-point Likert scale whether a certain item (e.g., “I really get involved with the feelings of the characters in a novel”) *does not describe [them] well* to *describes [them]very well*. Although the IRI consists of four scales, in practice, the scales are often merged to build indexes for cognitive empathy (*perspective taking & fantasy*) and affective empathy (*empathic concern & personal distress*). However, this use is criticized, e.g., by Chrysikou and Thompson (2015) who could not confirm the two-factor structure, but instead found that the initial four-factor model was superior. For this reason, we also employed another empathy questionnaire: The *Questionnaire of Cognitive and Affective Empathy* (QCAE; Reniers et al., 2011). The 31-item questionnaire includes items from the IRI, the *Empathy Quotient* (EQ; Baron-Cohen & Wheelwright, 2004), the *Hogan Empathy Scale* (HES; Hogan, 1969), as well as the empathy subscale of the *Impulsiveness-Venturesomeness-Empathy Inventory* (IVE; Eysenck & Eysenck, 1978) and is specifically designed to capture cognitive and affective empathy separately. Cognitive empathy includes the subscales *perspective taking* and *online simulation,* and affective empathy includes the subscales *emotion contagion*, *proximal responsivity* and *peripheral responsivity.* The 4-point Likert scale ranges from “strongly agree” to “strongly disagree” to statements such as “I get very upset when I see someone cry”. The questionnaire has shown good convergent and construct validity and its factor structure (two higher-order factors) could be confirmed (Reniers et al., 2011; Di Girolamo et al., 2019). In this sample, the internal consistency of the IRI scales ranged from α=.75 - α= .78; for the two cognitive empathy scales combined it was α= .73 and for the affective empathy scales combined α= .76; which can all be interpreted as acceptable. The QCAE scales on the other hand show acceptable to good internal consistency for the cognitive empathy scales (α= .77 and α=.86) as well as for cognitive empathy combined (α=.86); however, the affective empathy scales varied much more in their internal consistency from unacceptable (α=.47) for peripheral responsivity and questionable (α=.68) for proximal responsivity to acceptable (α=.76) for emotion contagion. Overall, the affective empathy scale showed an acceptable reliability (α=.76).

To assess adult attachment the *Experiences in Close Relationships* (ECR; Brennan et al., 1998) was used in a Swedish translation (Broberg & Granqvist, 2003). The widely-used self-report questionnaire consists of 36 items belonging to the dimensions *attachment anxiety* and *attachment avoidance*. Attachment anxiety is characterized by the exaggerated fear of being abandoned by a partner and corresponding measures to prevent abandonment (e.g., “I worry a fair amount about losing my partner”), whereas attachment avoidance is characterized by feeling uncomfortable being emotionally close to a partner and avoiding closeness (e.g., “I get uncomfortable when a romantic partner wants to be very close”). High scores on either or both of those scales indicate attachment insecurity. The participant is asked to indicate their agreement with a statement on a 7-point Likert scale (1 = strongly disagree – 7 = strongly agree). In this sample, the anxiety scale shows good (α=.86) and the avoidance scale excellent (α=.93) reliability.

In the posttest, the participants rated thirteen statements about their experiences and satisfaction with the trainings (e.g., “The training was very difficult”, “The training experiences will change my way of interacting with others” or “The training was fun”) on a scale from 0=strongly disagree to 5=strongly agree. The subjects were also asked for their feedback and suggestions for improvement in an open-ended question. This data is not presented as it simply should provide some subjective feedback to inform possible changes of the trainings for future studies.

**Trait/state questionnaires and ERA variables (results)**

*Group differences, regression analyses and correlations*

According to one-sample Wilcoxon signed rank tests, there were no differences between pre- and post-scores for the PANAS, IPANAT, KSS, or self-reported multimodal ERA. There was a slight but significant difference between pre- and postscores for self-reported micro expression ERA (*V* = 403, *p* < .03). *Supplementary Table 2* shows descriptive statistics and comparisons of the training groups. Before the post measurement, the multimodal training group reported less negative state affect in the PANAS than the micro expression training group (*p* = .01). To further investigate possible influences of implicit and explicit affective state on the ERA variables during pre- and post-measurement, we conducted (multiple) linear regression analyses. The result for the MICRO post measurement suggested a possible small influence of implicit affective state measured by the IPANAT (*F*(2, 64) = 5.31, *p*=.01, *adj. R^2^* = .12). All other regression analyses spoke against an influence of affective state on ERA. Since this was the only small influence of affective state on ERA and no significant differences in implicit state affect could be detected between the groups (see *Supplementary Table 2*), as well as not to complicate our analyses further, we decided against controlling for state affectivity in the ART ANOVAs. Before the post measurement, the multimodal training group reported lower self-reported ERA than the micro expression training group (*p* = .05), and the control training group (*p* = .05; Wilcoxon signed rank test, Holm adjusted).

*Supplementary Figure 1* displays a visualization of a Spearman’s correlation matrix (Holm adjusted) among the ERA and trait and state variables (questionnaires) for descriptive and exploratory purposes. There are medium to large correlations between the ERAM and MICRO variables irrespective of test time point, with the strongest correlations being between pre and posttest of the same ERA facet. Interestingly, the PECT pre score is only positively related to the ERAM pre/post scores; no or slightly negative correlations were found between PECT pre and MICRO pre/post scores. The PECT post score, was slightly negatively associated with pre/post scores for both ERAM and MICRO. The cognitive and affective empathy components of both empathy questionnaires (IRI and QCAE) were strongly positively related to one another (with the exception of cognitive empathy according to the QCAE and affective empathy according to the IRI). Further, the self-reports about multimodal ERA and micro expression ERA pre- and post-intervention were strongly positively correlated. A paired Wilcoxon signed rank test revealed that there was no difference between self-reported multimodal ERA in the pre- and the posttest (*V* = 499, *p* = .54); however, self-reported micro expression ERA did increase significantly (*V* = 403, *p* = .03).

Another correlational pattern could be seen for affective state. Pre and post scores of the affective state variables were strongly positively correlated. This pattern was found true for all four variables. The strongest negative correlations were found between sleepiness and explicit positive mood (PANAS) and cognitive empathy in both IRI and QCAE. The PECT scores also seemed to be stronger related to empathy as assessed by the two questionnaires than the other ERA variables. Correlations between self-reported multimodal ERA, self-reported micro expression ERA, ERAM and MICRO scores (pre/post) were very small. The PECT pre/post scores, on the other hand, showed substantial associations with the self-reports. The correlations between affective state and ERA were diverse. As only clear result, the positive IPANAT score during posttest was relatively strongly negatively related to all ERA variables.

**Trait/state questionnaires and ERA variables (discussion)**

The correlations between the ERAM and MICRO variables suggests that, even though no transfer effects for the trainings were found, those two competencies are somewhat related. Correlations decreased from pretest to posttest, suggesting a stronger specialization due to training. There were small negative associations between the PECT score in the posttest and the other two ERA tests (pre and post). This supports the result that the participants improved in multimodal and micro expression recognition abilities, but not in recognizing patient emotion cues.

The positive correlations between PECT and empathy scores could lead back to the fact that the PECT included both verbal and nonverbal emotional expressions from medical settings. It is plausible that a stronger accuracy for the featured patient’s emotional cues was associated with greater trait empathy. Noteworthily, in the QCAE, affective empathy seemed to have stronger correlations with all ERA measures than cognitive empathy, although cognitive empathy, per definition, consists of taking another person’s perspective mentally, oftentimes equated with emotion recognition. This pattern is less clear though when measuring empathy with the IRI. The strong pre−post correlations for the state affect variables suggest that the affectivity tests captured trait as well as state affect, something that is known from other studies. Sleepiness was negatively associated with positive affect and the empathy scores, most strongly with cognitive empathy. It could be cautiously interpreted that sleepiness led to diminished mood and empathic abilities. Nevertheless, there was no association between sleepiness and ERA in the pretest and only slight correlations in the posttest.

There was indication that affective state could have influenced the micro expression results in the posttest. However, by conducting several multiple regressions, the type I error probability was inflated and we concluded, that it was not worth complicating the analysis further by introducing a covariate into the ART ANOVAs. Another reason for deciding against controlling for implicit affective state was that there were no group differences in affective state and because this was the only time that affective state seemed to play a role. However, a small influence of positive affective state in the MICRO posttest cannot be ruled out completely and weakens the interpretability of the MICRO results.

The finding that the multimodal training group felt less explicitly negative than the micro expression training group before the posttest could be interpreted as them feeling better prepared for the posttest session. Still, they also estimated their own ERA to be lower than the other two groups did, which could contradict this interpretation. Feeling less confident about one’s multimodal ERA could display a negative aspect of repeated ERA training. To be continuously confronted with evaluations about one’s ERA could lead to diminished confidence in one’s own ability. The motivational aspects of ERA training should be investigated in future studies.

Interestingly, there were small (positive, as well as negative) correlations between *self-reported* multimodal/micro expression ERA and *objective* multimodal/micro expression ERA. Furthermore, the PECT pre- and post-scores were relatively strongly associated with subjective multimodal ERA and micro expression ERA skills. One way to hypothesize is that the PECT, as containing verbal and nonverbal ERA skills, was more in line with the participants real life experiences with and understanding of ERA and that this contributed to a stronger positive association. The questions about subjective ERA, even though concerning multimodal and micro expression recognition specifically, did possibly capture more of a general verbal and nonverbal ERA skill. Another interpretation is that the individuals were simply not very accurate at judging their own multimodal and micro expression recognition accuracy. A further hypothesis that needs to be tested in future research.

**References (supplementary)**

Baron-Cohen, S., & Wheelwright, S. (2004). The empathy quotient: An investigation of adults with Asperger syndrome or high functioning autism, and normal sex differences. *Journal of Autism and Developmental Disorders*, *34*, 163–175. <https://doi.org/10.1023/b:jadd.0000022607.19833.00>

Brennan, K. A., Clark, C. L., & Shaver, P. R. (1998). Self-report measurement of adult attachment: An integrative overview. In J. A. Simpson & Rhodes, W.S. (Eds.), *Attachment theory and close relationships* (pp. 46–76). New York: Guilford Press.

Broberg, A. G., & Granqvist, P. (2003). Erfarenhet av nära relationer. Unpublished material.

Chrysikou, E. G., & Thompson, W. J. (2016). Assessing Cognitive and Affective Empathy Through the Interpersonal Reactivity Index: An Argument Against a Two-Factor Model. *Assessment*, *23*(6), 769–777. <https://doi.org/10.1177/1073191115599055>

Di Girolamo, M., Giromini, L., Winters, C. L., Serie, C. M. B, & de Ruiter, C. (2019) The Questionnaire of Cognitive and Affective Empathy: A Comparison Between Paper-and-Pencil Versus Online Formats in Italian Samples, *Journal of Personality Assessment*, *101*(2), 159–170. <https://doi.org/10.1080/00223891.2017.1389745>

Eysenck, S. B. G., & Eysenck, H. J. (1978). Impulsiveness and venturesomeness: Their position in a dimensional system of personality description. Psychological Reports, 43(3), 1247-1255. [doi: 10.2466/PR0.43.7.1247-1255](http://ammonsscientific.com/link.php?N=16283)

Hogan, R. (1969). Development of an empathy scale. Journal of Consulting and Clinical Psychology, 33(3), 307–316. [https://doi.org/10.1037/h0027580](https://psycnet.apa.org/doi/10.1037/h0027580)

Quirin, M., Kazén, M., & Kuhl, J. (2009). When nonsense sounds happy or helpless: The Implicit Positive and Negative Affect Test (IPANAT). *Journal of Personality and Social Psychology, 97(3)*, 500–516. <http://doi.org/10.1037/a0016063>

Quirin, M., Wróbel, M., Norcini Pala, A., Stieger, S., Brosschot, J., Kazén, M., Hicks, J. A., Mitina, O., Shanchuan, D., Lasauskaite, R., Silvestrini, N., Steca, P., Padun, M. A., & Kuhl, J. (2018). A cross-cultural validation of the Implicit Positive and Negative Affect Test (IPANAT): Results from ten countries across three continents. European Journal of Psychological Assessment, 34(1), 52–63. [https://doi.org/10.1027/1015-5759/a000315](https://psycnet.apa.org/doi/10.1027/1015-5759/a000315)

Watson, D., Clark, L. A., & Tellegen, A. (1988). Development and validation of brief measures of positive and negative affect: The PANAS scales. Journal of Personality and Social Psychology, 54(6), 1063–1070. [https://doi.org/10.1037/0022-3514.54.6.1063](https://psycnet.apa.org/doi/10.1037/0022-3514.54.6.1063)

Wei, T. & Simko, V. (2017). *R package "corrplot": Visualization of a Correlation Matrix* (Version 0.84). Available from <https://github.com/taiyun/corrplot>

Åkerstedt, T., & Gillberg, M. (1990). Subjective and objective sleepiness in the active individual. International Journal of Neuroscience, 52(1-2), 29–37. [https://doi.org/10.3109/00207459008994241](https://psycnet.apa.org/doi/10.3109/00207459008994241)

**Supplementary Figure 1**

*Visualization of Spearman’s correlation matrix for the ERA and questionnaire variables (Holm adjusted)*


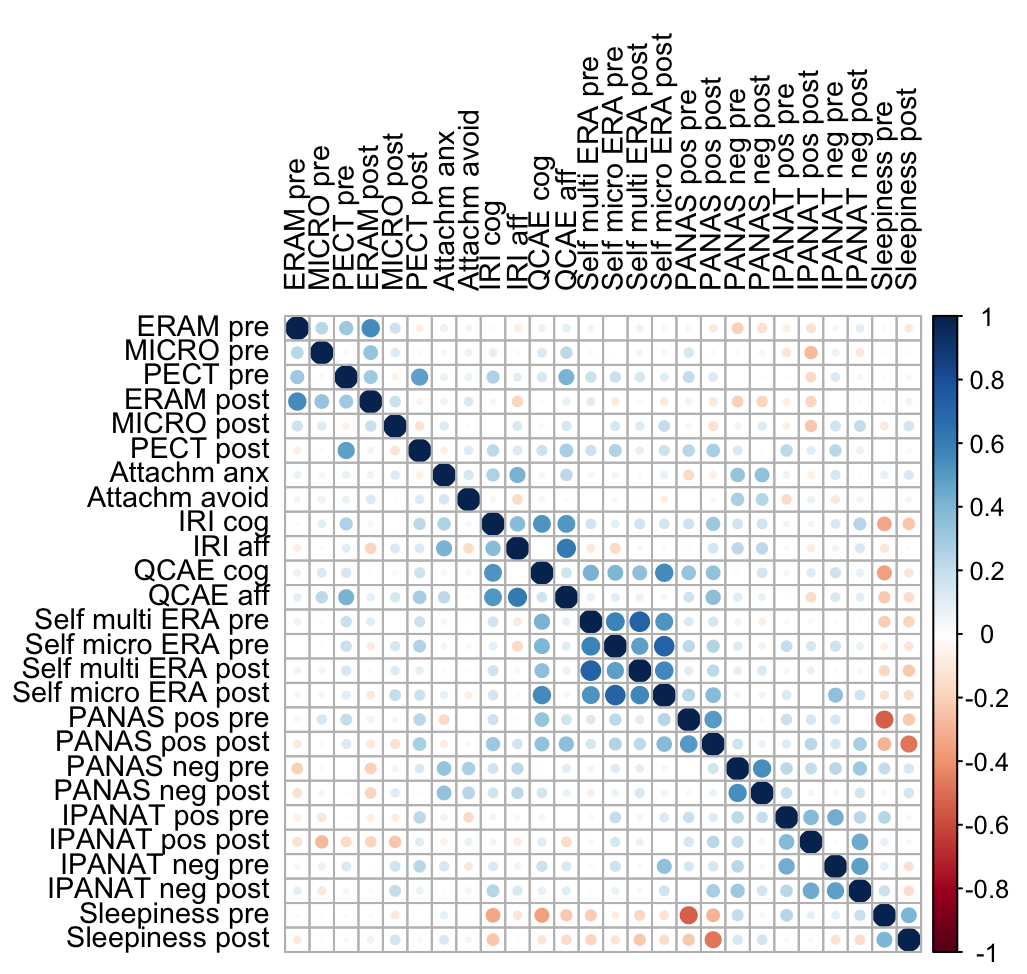


*Note*. Positive correlations are displayed in blue, negative correlations in red. The size of a circle and intensity of color are proportional to the correlation coefficients (Spearman’s r_s_). The legend on the right provides information about color intensity and magnitude of association (correlation coefficients). Only significant (*p* < .05) are displayed, blank fields represent that there is no significant correlation between variables.

**Supplementary Table 1**

*Descriptive statistics (means, standard deviations) for single emotions of the ERA measures (pre/post) per training group*

|  | **Multimodal Training** | | **Micro expression training** | | **Control Training** | |
| --- | --- | --- | --- | --- | --- | --- |
| **Emotion** | ***pre*** | ***post*** | ***pre*** | ***post*** | ***pre*** | ***post*** |
| ***ERAM*** |  |  |  |  |  |  |
| **Anger** | .58  (.17) | .71  (.21) | .65  (.15) | .69  (.15) | .65  (.18) | .69  (.16) |
| **Anxiety** | .18  (.14) | .23  (.16) | .15  (.13) | .24  (.19) | .28  (.22) | .34  (.25) |
| **Despair** | .22  (.18) | .42  (.22) | .25  (.18) | .27  (.17) | .35  (.19) | .36  (.19) |
| **Disgust** | .35  (.23) | .63  (.23) | .39  (.25) | .43  (.24) | .46  (.24) | .52  (.22) |
| **Fear** | .29  (.20) | .45  (.22) | .31  (.17) | .31  (.16) | .32  (.16) | .37  (.22) |
| **Interest** | .42  (.20) | .52  (.23) | .34  (.18) | .42  (.18) | .44  (.17) | .46  (.20) |
| **Irritation** | .41  (.23) | .44  (.20) | .38  (.22) | .43  (.21) | .37  (.16) | .47  (.23) |
| **Joy** | .35  (.20) | .57  (.20) | .34  (.18) | .46  (.17) | .37  (.17) | .48  (.17) |
| **Pleasure** | .41  (.25) | .68  (.21) | .55  (.16) | .60  (.23) | .53  (.21) | .56  (.17) |
| **Pride** | .41  (.21) | .50  (.17) | .28  (.15) | .46  (.23) | .35  (.15) | .50  (.18) |
| **Relief** | .40  (.22) | .62  (.28) | .44  (.21) | .47  (.21) | .47  (.21) | .51  (.21) |
| **Sadness** | .28  (.24) | .35  (.23) | .24  (.25) | .32  (.23) | .37  (.22) | .38  (.25) |
| ***MICRO*** |  |  |  |  |  |  |
| **Anger** | .74  (.40) | .71  (.42) | .48  (.47) | .77  (.42) | .53  (.46) | .70  (.40) |
| **Contempt** | .52  (.38) | .66  (.34) | .40  (.36) | .74  (.37) | .42  (.38) | .61  (.38) |
| **Disgust** | .49  (.31) | .49  (.31) | .39  (.25) | .72  (.32) | .38  (.30) | .46  (.30) |
| **Fear** | .60  (.40) | .48  (.44) | .67  (.35) | .79  (.38) | .67  (.37) | .78  (.27) |
| **Joy** | .52  (.41) | .73  (.32) | .35  (.36) | .84  (.30) | .51  (.39) | .50  (.41) |
| **Sadness** | .59  (.45) | .69  (.38) | .76  (.26) | .81  (.28) | .61  (.32) | .70  (.32) |
| **Surprise** | .35  (.36) | .48  (.39) | .36  (.31) | .57  (.40) | .37  (.41) | .59  (.41) |
| ***PECT*** |  |  |  |  |  |  |
| **Anger** | .46  (.18) | .50  (.18) | .40  (.17) | .45  (.22) | .48  (.12) | .52  (.15) |
| **Anxiety** | .32  (.17) | .36  (.19) | .32  (.16) | .36  (16) | .38  (.21) | .40  (.21) |
| **Confusion** | .45  (.18) | .48  (.19) | .43  (.18) | .52  (.14) | .46  (.20) | .55  (.18) |
| **Happiness** | .57  (.15) | .55  (.21) | .51  (.23) | .62  (.12) | .57  (.11) | .56  (.15) |
| **Neutral** | .24  (.16) | .35  (.15) | .25  (.17) | .26  (.15) | .27  (.15) | .36  (.16) |
| **Sadness** | .46  (.17) | .57  (.23) | .44  (.14) | .46  (.16) | .49  (.19) | .51  (.22) |

Note. *N* = 67. PECT data for one participant (multimodal training group) lost due to technical reasons. The ERA scores (range 0-1) are presented as unbiased hitrates (H_u_).

**Supplementary Table 2**

*Descriptive statistics (means, standard deviations, 95% confidence intervals) and comparisons of the three groups for the questionnaire variables*

| **Measures** | ***N*** | **Multimodal training** | **Micro expression training** | **Control training** | **Overall** | ***F* / χ^2^** | **η^2^ / ε^2^** |
| --- | --- | --- | --- | --- | --- | --- | --- |
| Attachment anxiety | 67 | 3.77 (.92)  [3.35, 4.19] | 4.27 (.94)  [3.87, 4.68] | 4.14 (1.00)  [3.71, 4.57] | 4.07 (.96)  [3.84, 4.31] | *F*(2,64) = 1.61 (*p* = .21) | η^2^ = .05 |
| Attachment avoidance | 67 | 2.88 (1.21)  [2.33, 3.43] | 2.54 (.99)  [2.11, 2.97] | 2.81 (1.12)  [2.33, 3.30] | 2.74 (1.1)  [2.47, 3.01] | χ^2^(2) = 0.82 (p = .66) | ε^2^ = .01 |
| IRI cognitive empathy | 67 | 2.82 (.52)  [2.58, 3.06] | 2.78 (.55)  [2.55, 3.02] | 2.73 (.46)  [2.53, 2.92] | 2.78 (.5)  [2.65, 2.90] | *F*(2,64) = 0.19 (*p* = .83) | η^2^ = .01 |
| IRI affective empathy | 67 | 2.18 (.61)  [1.91, 2.46] | 2.48 (.48)  [2.27, 2.68] | 2.37 (.46)  [2.17, 2.56] | 2.35 (.52)  [2.22, 2.47] | χ^2^(2) = 2.10 (p = .35) | ε^2^ = .03 |
| QCAE cognitive empathy | 67 | 3.16 (.31)  [3.02, 3.30] | 3.13 (.39)  [2.96, 3.30] | 3.15 (.29)  [3.02, 3.27] | 3.14 (.33)  [3.06, 3.22] | *F*(2,64) = 0.04 (*p* = .96) | η^2^ = .00 |
| QCAE affective empathy | 67 | 2.98 (.49)  [2.76, 3.20] | 3.11 (.36)  [2.95, 3.26] | 3.00 (.42)  [2.82, 3.18] | 3.03 (.42)  [2.93, 3.13] | *F*(2,64) = 0.57 (*p* = .57) | ε^2^ = .02 |
| Sleepiness (pre) | 67 | 4.90 (2.02)  [3.98, 5.83] | 4.53 (1.20)  [3.92, 4.95] | 4.61 (1.73)  [3.86, 5.35] | 4.64 (1.66)  [4.24, 5.05] | *F*(2,64) = 0.44 (*p* = .65) | η^2^ = .01 |
| Sleepiness (post) | 67 | 4.52 (1.63)  [3.78, 5.27] | 4.48 (1.62)  [3.78, 5.18] | 4.00 (1.21)  [3.48, 4.52] | 4.33 (1.49)  [3.96, 4.69] | χ^2^(2) = 1.41 (p = .49) | ε^2^ = .02 |
| PANAS positive (pre) | 67 | 3.16 (.67)  [2.86, 3.47] | 2.91 (.71)  [2.60, 3.22] | 3.11 (.96)  [2.69, 3.52] | 3.06 (.79)  [2.86, 3.25] | *F*(2,64) = 0.63 (*p* = .54) | η^2^ = .02 |
| PANAS positive (post) | 67 | 2.95 (.62)  [2.67, 3.24] | 2.82 (.78)  [2.49, 3.16] | 2.85 (.77)  [2.52, 3.18] | 2.87 (.72)  [2.70, 3.05] | χ^2^(2) = 0.32 (p = .85) | ε^2^ = .00 |
| PANAS negative (pre) | 67 | 1.29 (.35)  [1.13, 1.45] | 1.44 (.43)  [1.25, 1.63] | 1.37 (.37)  [1.21, 1.53] | 1.37 (.38)  [1.27, 1.46] | χ^2^(2) = 2.92 (p = .23) | ε^2^ = .04 |
| PANAS negative (post) | 67 | 1.18 (.34)  [1.03, 1.33] | 1.52 (.52)  [1.30, 1.75] | 1.31 (.50)  [1.09, 1.52] | 1.34 (.48)  [1.23, 1.46] | χ^2^(2) = 9.86 (p = .01)** | ε^2^ = .15 |
| IPANAT positive (pre) | 66 | 1.96 (.44)  [1.76, 2.15] | 2.01 (.31)  [1.88, 2.15] | 2.05 (.55)  [1.81, 2.28] | 2.01 (.44)  [1.90, 2.12] | *F*(2,63) = 0.22 (*p* = .80) | η^2^ = .01 |
| IPANAT positive (post) | 67 | 1.98 (.38)  [1.80, 2.15] | 1.92 (.30)  [1.80, 2.05] | 1.94 (.44)  [1.75, 2.13] | 1.95 (.37)  [1.86, 2.04] | *F*(2,64) = 0.12 (*p* = .89) | η^2^ = .00 |
| IPANAT negative (pre) | 66 | 1.91 (.32)  [1.77, 2.06] | 2.05 (.41)  [1.87, 2.23] | 1.87 (.36)  [1.72, 2.03] | 1.95 (.37)  [1.86, 2.04] | *F*(2,63) = 1.44 (*p* = .25) | η^2^ = .04 |
| IPANAT negative (post) | 67 | 1.96 (.40)  [1.77, 2.14] | 2.11 (.42)  [1.93, 2.29] | 1.96 (.43)  [1.77, 2.15] | 2.01 (.42)  [1.91, 2.11] | *F*(2,64) = 1.03 (*p* = .37) | η^2^ = .03 |
| Multimodal ERA self-report (pre) | 67 | 3.80 (.49)  [3.57, 4.02] | 3.83 (.70)  [3.52, 4.13] | 3.98 (.43)  [3.79, 4.17] | 3.87 (.55)  [3.73, 4.00] | *F*(2,64) = 0.69 (*p* = .51) | η^2^ = .02 |
| Multimodal ERA self-report (post) | 64 | 3.58 (.52)  [3.35, 3.82] | 3.96 (.53)  [3.73, 4.19] | 3.97 (.48)  [3.76, 4.17] | 3.84 (.53)  [3.71, 3.97] | χ^2^(2) = 7.38 (p = .02)* | ε^2^ = .11 |
| Micro expression ERA self-report (pre) | 67 | 2.87 (.74)  [2.53, 3.21] | 3.00 (.78)  [2.66, 3.34] | 3.08 (.55)  [2.84, 3.31] | 2.99 (.69)  [2.82, 3.15] | *F*(2,64) = 0.50 (*p* = .61) | η^2^ = .02 |
| Micro expression ERA self-report (post) | 64 | 3.05 (.67)  [2.74, 3.35] | 3.15 (.66)  [2.87, 3.44] | 3.15 (.71)  [2.84, 3.45] | 3.12 (.67)  [2.95, 3.29] | *F*(2,61) = 0.16 (*p* = .85) | η^2^ = .01 |

*Note*. Missing data for IPANAT (pre) and multimodal ERA/micro expression ERA self-report (post) due to technical reasons.

**p* < .05, ***p* < .01, ****p* < .001.
